# Nanoengineered cargo with targeted *in vivo Foxo3* gene editing modulated mitophagy of chondrocytes to alleviate osteoarthritis

**DOI:** 10.1016/j.apsb.2024.12.008

**Published:** 2024-12-14

**Authors:** Manyu Chen, Yuan Liu, Quanying Liu, Siyan Deng, Yuhan Liu, Jiehao Chen, Yaojia Zhou, Xiaolin Cui, Jie Liang, Xingdong Zhang, Yujiang Fan, Qiguang Wang, Bin Shen

**Affiliations:** a National Engineering Research Center for Biomaterials, College of Biomedical Engineering, Sichuan University, Chengdu 610064, China; b Orthopedics Research Institute, Department of Orthopedics, West China Hospital, Sichuan University, Chengdu 610041, China; c Institute of Rocket Force Medicine, State Key Laboratory of Trauma, Burns and Combined Injury, Third Military Medical University (Army Medical University), Chongqing 400038, China; d The Third Affiliated Hospital of Jinzhou Medical University, Jinzhou 121000, China; e Animal Laboratory Center of West China Hospital, West China Hospital, Sichuan University, Chengdu 610041, China; f Christchurch Regenerative Medicine and Tissue Engineering (CReaTE) Group, School of Medicine, the Chinese University of Hong Kong, Shenzhen 518172, China; g Department of Orthopedic Surgery & Musculoskeletal Medicine, Centre for Bioengineering & Nanomedicine, University of Otago, Christchurch 8140, New Zealand; h Sichuan Testing Center for Biomaterials and Medical Devices, Sichuan University, Chengdu 610064, China

**Keywords:** Osteoarthritis, Mitophagy, Nanoengineered cargo, *In vivo Foxo3* gene editing, Cartilage regeneration, Injectable hydrogel microspheres, PINK1/Parkin pathway, CRISPR/Cas9 gene editing

## Abstract

Mitochondrial dysfunction in chondrocytes is a key pathogenic factor in osteoarthritis (OA), but directly modulating mitochondria *in vivo* remains a significant challenge. This study is the first to verify a correlation between mitochondrial dysfunction and the downregulation of the *FOXO3* gene in the cartilage of OA patients, highlighting the potential for regulating mitophagy *via FOXO3* gene modulation to alleviate OA. Consequently, we developed a chondrocyte-targeting CRISPR/Cas9-based *FOXO3* gene-editing tool (FoxO3) and integrated it within a nanoengineered ‘truck’ (NETT, FoxO3-NETT). This was further encapsulated in injectable hydrogel microspheres (FoxO3-NETT@SMs) to harness the antioxidant properties of sodium alginate and the enhanced lubrication of hybrid exosomes. Collectively, these FoxO3-NETT@SMs successfully activate mitophagy and rebalance mitochondrial function in OA chondrocytes through the *Foxo3* gene-modulated PINK1/Parkin pathway. As a result, FoxO3-NETT@SMs stimulate chondrocytes proliferation, migration, and ECM production *in vitro*, and effectively alleviate OA progression *in vivo*, demonstrating significant potential for clinical applications.

## Graphical abstract

FoxO3-NETT@SMs targeting-regulated *Foxo3* gene *in vivo* to modulate mitophagy for osteoarthritis therapy.

**Figure:**
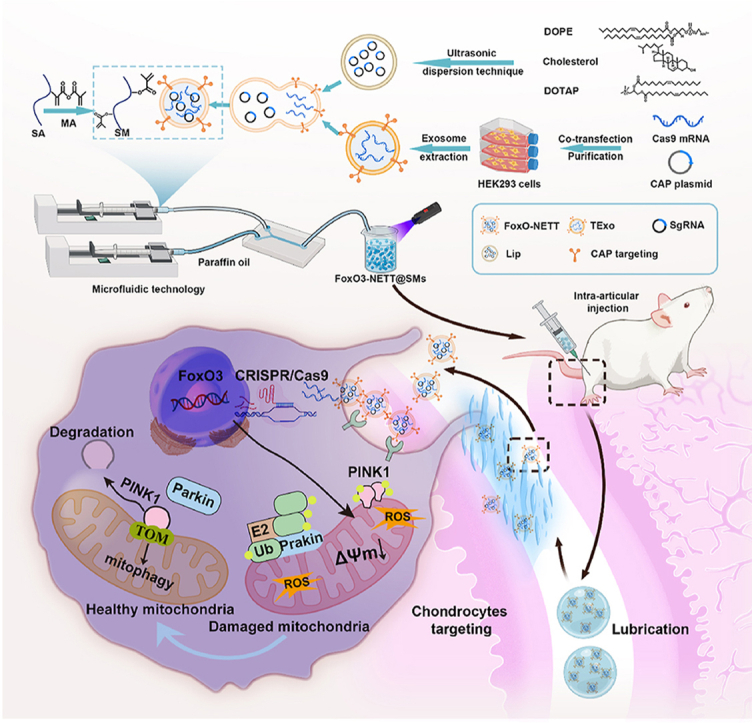

## 1 Introduction

Osteoarthritis (OA) is an age-related disease characterized by the deterioration of chondrocytes and their intracellular organelles, with mitochondrial dysfunction being a key pathogenic factor^1^^,^^2^. In OA, chondrocytes’ mitochondrial structure and function undergo significant changes due to pathological alterations in the osteoarthritic microenvironment^3^. An imbalance in mitochondrial dynamics, marked by impaired autophagic regulation, leads to the accumulation of aged and damaged mitochondria^4^. The inability to efficiently eliminate these mitochondria results in the release of apoptotic factors and proteins, triggering a cascade that leads to mitochondrial and chondrocyte apoptosis, contributing to cartilage degeneration^5^^,^^6^. Therefore, rejuvenating mitophagy and restoring mitochondrial dynamics could play a crucial role in revitalizing chondrocytes, potentially enhancing cartilage repair and regeneration.

The *FOXO3* gene, a key transcription factor in the Forkhead box O (FoxO) family, plays crucial roles in antioxidant defense, stress response, and cellular metabolism^7^^,^^8^. Previous studies by Akasaki et al.^9^ and Fisch et al.^10^ have detected downregulated expression of the *FOXO3* gene in OA chondrocytes in both patients and mouse models. And *FOXO3* was particularly regarded as a primary positive regulator of mitophagy^11^. Recent studies highlight *FOXO3*'s protective roles in mitigating chondrocyte damage in OA. For instance, *Foxo3* maintains homeostasis and protects against meniscus damage due to mechanical overuse and aging^12^. Cartilage-specific deletion of *Foxo3* leads to irregular cartilage maturation and exacerbates OA, along with significantly reduced expression levels of genes related to chondrocyte autophagy and anti-oxidative stress^13^. *Foxo3* also suppresses ferroptosis in OA chondrocytes, a process leading to cell death and extracellular matrix (ECM) disruption^14^. Together, these findings suggest a potential correlation between *Foxo3* activation and OA progression. However, to consider *FOXO3* as a therapeutic target for OA, it's crucial to determine *FOXO3* expression alterations during OA progression, particularly in the context of mitochondrial dysfunction and chondrocytes' function *via FOXO3* modulation. In addition, exploring the specific mechanisms underlying *FOXO3*'s regulation in mitochondrial autophagy and dynamic stability is crucial for alleviating OA progression.

The complexity of the intracellular environment and cell membrane barrier make targeted regulation of mitochondria challenging, and direct gene interventions often have limited *in vivo* efficiency^15^, ^16^, ^17^, ^18^. However, the advent of gene-editing tool, CRISPR/Cas9 gene editing technology, provides the possibility to precisely regulate specific expression associated with mitophagy^19^. For *in vivo* CRISPR/Cas9 editing, hybrid exosomes, synthesized through the fusion of exosomes with liposomes, present an effective delivery vehicle for CRISPR/Cas9 tools due to their safety, biocompatibility, and efficient cell membrane penetration^20^, ^21^, ^22^, ^23^, ^24^, ^25^. This makes them ideal for activating the *Foxo3* gene in OA chondrocytes, regulating mitochondrial function, and restoring balance in mitochondrial dynamics. This strategy holds significant potential as a novel treatment modality for OA.

Moreover, the pathological microenvironment of cartilage contributes to tissue degeneration and OA progression^26^^,^^27^. Maintaining homeostasis in this environment is crucial to prevent further OA development^28^. Sodium alginate (SA), a natural polysaccharide, has antioxidant properties that enhance the osteoarthritic microenvironment and promote the survival and functionality of OA-affected chondrocytes^29^, ^30^, ^31^. Grafting methyl methacrylate groups onto SA allows nanoscale hybrid exosomes to anchor within the SA gel network, ensuring prolonged retention at the joint site^32^. When combined with hybrid exosomes, the hydrogel exhibits superior lubrication performance, protecting against wear, alleviating pain, and reducing cartilage degradation^33^, ^34^, ^35^, ^36^. This helps maintain homeostasis in the cartilage microenvironment during OA progression. It not only alleviates intracellular oxidative stress but also enhances joint lubrication, reducing inflammation during movement.

**Scheme 1:**
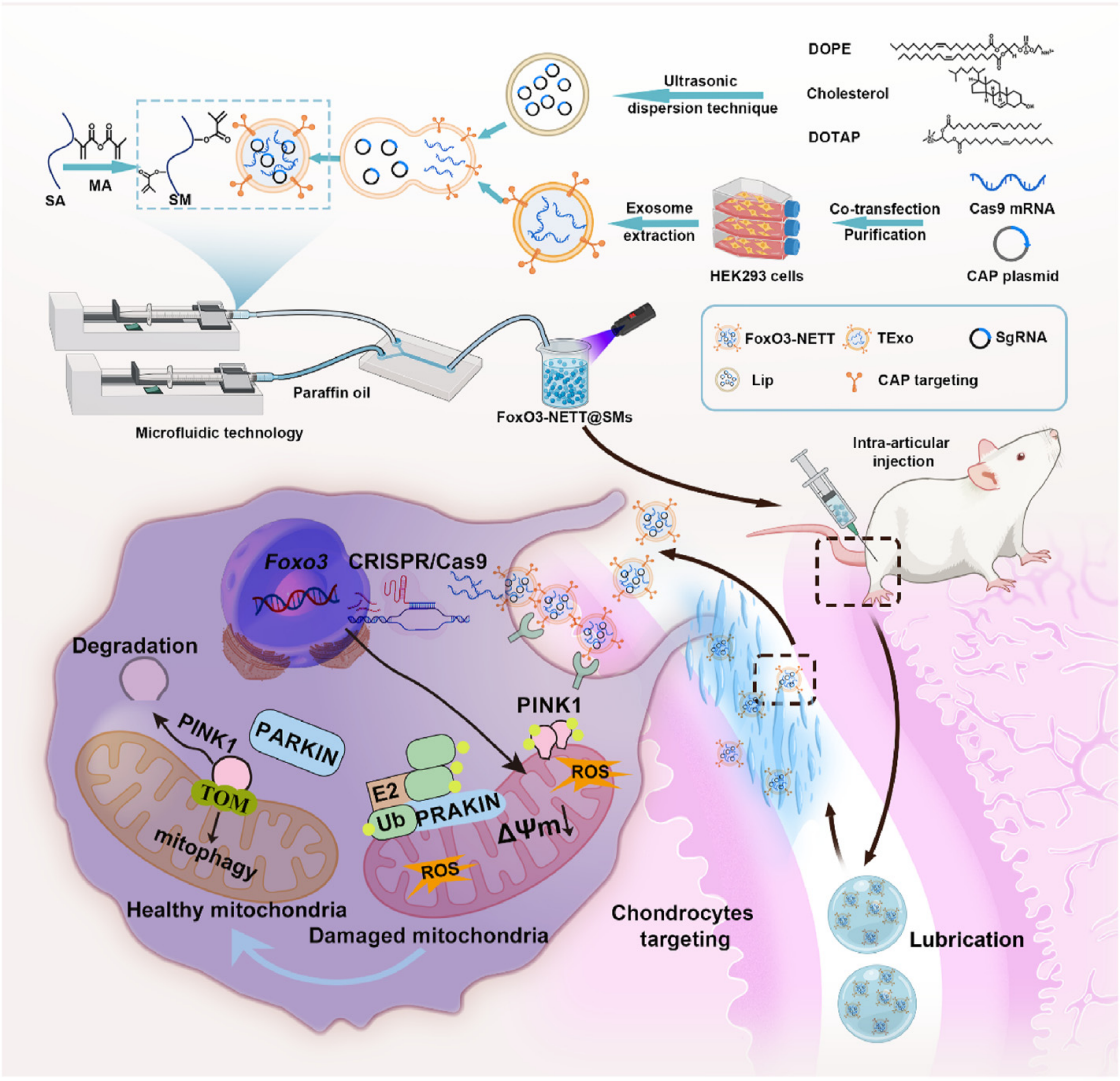
Schematic of FoxO3-NETT@SMs targeting-regulated *Foxo3* gene *in vivo* to modulate mitochondrial dynamics for osteoarthritis therapy.

This study is the first to investigate the relationship between mitochondrial dysfunction and *FOXO3* gene expression in human cartilage tissue across various OA stages, corroborated using a rat OA model. Based on these findings, we propose a strategy to mitigate OA progression by enhancing mitochondrial autophagy and restoring mitochondrial function *via FOXO3* gene modulation (Scheme 1). Consequently, a CRISPR/Cas9-based *FOXO3*-targeted gene-editing tool was incorporated into a chondrocyte-targeting nanoengineered ‘truck’, by fusing SgFoxO3 plasmid-enriched liposomes with Cas9 protein-containing chondrocyte-targeting exosomes. The resulting FoxO3-NETTs were anchored within a methacrylic acid-grafted sodium alginate and encapsulated into uniform hydrogel microspheres, forming FoxO3-NETT@SMs. The efficacy of FoxO3-NETT@SMs in activating the *FOXO3* gene towards the mitochondrial regulation was firstly validated. Subsequently, the effects of FoxO3-NETT@SMs on OA chondrocytes were evaluated *in vitro*, including cell viability, morphology, proliferation, migration, and matrix formation. Lastly, the therapeutic potential of FoxO3-NETT@SMs in a rat OA model were assessed, with specific mechanisms elucidated through transcriptomic analysis. Together, this strategy paves the way for advanced use of CRISPR-based precision gene therapy to target mitochondria in OA management.

## 2 Materials and methods

### 2.1 Obtaining and examining knee-joint tissue in human and rats

The normal human knee cartilage specimens used in this study were obtained from lower limb amputees without any symptoms of OA. The human OA specimens used in this study were obtained from late-stage OA patients undergoing total knee arthroplasty. Sichuan University's West China Hospital Biomedical Ethics Committee approved this study (2022(200)). We randomly assigned 220–230 g male Sprague Dawley (SD) rats to a sham group and to an OA group for the collection of knee-joint tissue from rats. The sham group was used to obtain normal knee cartilage samples. The OA specimens were collected from rats after DMM surgery at 0, 2, 4, 6, 8, 10, and 12 weeks. All experimental procedures were executed according to the protocols approved by Sichuan University Animal Care and Use Committee (Protocol Number KS2020330).

For histological staining analysis, human knee cartilage tissues or rat knee tissues were harvested and immersed in 4% paraformaldehyde overnight. Frozen and paraffin sections were prepared and incubated with FOXO3 antibodies for immunofluorescence and immunohistochemistry staining. The semi-quantitative analysis of immunohistochemical or immunofluorescence intensity of FOXO3 was quantified using Image J software (National Institutes of Health, Bethesda, USA).

For ultrastructural analysis, human knee cartilage tissues were treated with 3% glutaraldehyde and then placed in embedding material. Methylene blue was used for stained semi-thin sections, and uranyl acetate and lead citrate were used for stained ultrathin sections. The sections were examined under a Transmission Electron Microscope (TEM, JEOL Ltd., JEM-1400-FLASH, Tokyo, Japan).

TRIzol (Invitrogen, 15596026CN, Carlsbad, CA, USA) was used to extract total RNA from human knee cartilage tissues or rat knee tissues for RT-PCR analysis. An iScript cDNA (Bio-Rad, 1708890, Hercules, CA, USA) Synthesis Kit was used for reverse transcription. The cDNAs were quantified by a CFX96 RT-PCR detection system (Bio-Rad CFX Manager Real-Time PCR System, Hercules, CA, USA) and Primers were listed in Supporting Information Table S1 and Table S2. Using the comparative cycle threshold (CT) method (ΔΔCT method), all experiments were performed in triplicate, and relative mRNA expression was calculated.

For mitochondrial membrane potential detection, the JC-1 Mitochondrial Membrane Potential Assay Kits (Solarbio, M8650, Beijing, China) were used to detect mitochondrial membrane potential in human chondrocytes obtained from human knee cartilage according to operating instructions. Using an Olympus fluorescent microscope (Olympus, IX83, Tokyo, Japan), fluorescent signals were recorded.

### 2.2 Preparation and characterization of the FoxO3-NETT

The *Foxo3* gene activation editing tools were constructed by Starfish Biological Technology Co., Ltd. using a sgRNA-FoxO3 plasmid and a synthetic Cas9 plasmid. Supporting Information Figs. S1 and S2 illustrate the profiles of both plasmids. The nanoengineered “truck” specifically targeting chondrocytes was prepared by membrane fusion between exosomes and liposomes. The exosomes were derived from HEK293 cells (the Cell Bank of the Chinese Academy of Sciences, Shanghai, China) according to the gradient centrifugation method, which were transfected with the CAP/EGFP-Lamp2b plasmid (encoding cartilage-affinity peptide) and Cas9 plasmid (expressing fusion Cas9 protein). To observe the morphology of exosomes, we utilized TEM (FEI, Tecnai G2 F20 S-TWIN, Hillsboro, Oregon, USA). Additionally, we employed dynamic light scattering (DLS, Malvern Nano-ZS, Malvern, UK) to detect the size distribution of exosomes. Using the ultrasonic dispersion method, liposomes were prepared^37^. Briefly, the mixture solution of DOPE, DOTAP, and cholesterol (2:2:1, *w*/*w*; solvent: CHCl_3_:CH_3_OH = 2:1, *w*/*w*) was sonicated for 5 min, evaporated at 60 °C until all solvents had evaporated, and filtered by a 0.45 μm filter (Millipore, Darmstadt, Germany). Subsequently, a gene pulser Xcell (Bio-Rad, CA, USA) was used to electroporate different amounts of SgRNA vector plasmid (0 or 5 μg) into the liposomes. The liposomes were detected by TEM and DLS for determining morphology, size distribution, and zeta potential. The mixture of exosomes and liposomes loaded with different amounts of sgRNA were incubated at 37 °C for 12 h, resulting in the formation of NETT and FoxO3-NETT. DLS and TEM were used to measure NETT and FoxO3-NETT. Efficacy of plasmid loading and FoxO3 gene editing of NETT and FoxO3-NETT were determined by PCR analysis.

### 2.3 Preparation and characterization of injectable FoxO3-NETT@SMs

The synthesis of SM was conducted as follows: Briefly, 1 g of SA was dissolved in 100 mL of PBS solution, followed by the addition of excess methyl acrylate (5-fold molar excess; Aladdin, China). The reaction mixture was then stirred continuously for 48 h at 4 °C, pH 8.0. Afterward, the reaction solution was dialyzed and subsequently freeze-dried to obtain white SM sponges. Characterization analysis of SM was performed using ^1^H NMR (600 MHz, Bruker, Germany).

A 2.5 % (*w*/*w*) mixture of SM-FoxO3-NETT-photo-initiator (40:6:4, *w*/*w*/*w*) was mixed into a microfluidic oil containing 95% (*w*/*w*) paraffin oil and 5% (*w*/*w*) Span 80. This mixture was used to form pre-gel droplets at the flow-focusing junction of the microfluidic device. The formed droplets were then crosslinked by exposure to ultraviolet (UV) light. Afterwards, the crosslinked microspheres were thoroughly washed and stored in PBS for further use. The bright-field microscope (ZEISS, LSM800, Oberkochen, Germany) was employed to observe the morphology and size of FoxO3-NETT@SM. To verify the successful integration of FoxO3-NETT into the hybrid hydrogel microspheres (SMs), FoxO3-NETT was labeled with DiO dye, and then combined with the SM matrix to produce hybrid SMs. Subsequent visualization of the DiO-labeled FoxO3-NETT within the SMs was conducted using a confocal laser scanning microscope (ZEISS, CLSM, LSM 880, Oberkochen, Germany).

During degradation testing, three groups of samples were immersed in PBS (pH = 7.4) and vibrated at 37 °C and 60 rpm. Each sample's residual weight was determined at predetermined time points by subtracting the supernatant and adding fresh solution every day. The testing of drugs encapsulated and released was conducted, the release curve of the encapsulated protein was assessed through the following procedure: Samples from three distinct groups were positioned in a *trans*-well with 8 μm pores (Corning, USA), submerged in PBS at 37 °C, and subjected to vibration at 80 rpm until the completion of drug release. In accordance with the instructions in the instruction manual (BCA Protein Quantification Kit, ThermoFisher, US), the solution containing release medium was collected at predefined intervals for protein quantification. Subsequently, the collected medium was replaced with an equivalent volume of PBS. The friction and wear test are performed by a reciprocating high-frequency tester using an 8-mm polyethylene sphere and a stainless-steel disk as surfaces (MGW-001, Jinan Yihua Tribology Testing Technology Co., Ltd., China). In this experiment, we set the oscillation amplitude at 4 mm and the frequency at 1 Hz. A volume of 1 mL of either FoxO3-NETT@SMs or SMs suspension (60 mg/mL) was initially applied to the lower surface, and the test proceeded for 300 s. The effectiveness of lubrication of SMs and FoxO3-NETT@SMs were assessed and compared under uniform experimental conditions.

### 2.4 Construction of the IL-1β-induced OA chondrocytes model

Our experiments were performed with third-passage chondrocytes isolated from knee joint cartilage of 7-day-old SD rats. The adhesive third-passage chondrocytes were cultured in culture medium containing 10 ng/mL of IL-1*β* for 24 h. Subsequently, *in vitro* biological experiments were conducted using IL-1*β*-induced chondrocytes.

### 2.5 Assessment of enhanced uptake efficiency of FoxO3-NETT by chondrocytes *in vitro*

To assess the enhanced uptake efficiency of FoxO3-NETT *in vitro*, DiO labeling was performed on Exo, TExo, and FoxO3-NETT following the manufacturer's protocol. Chondrocytes were seeded in 24-well plates at a density of 2 × 10^4^ cells per well. After co-incubating with labeled hybrid exosomes in a cell culture incubator for 8 h, the samples were analyzed using CLSM. Quantitative analysis of fluorescence intensity was performed using ImageJ software.

### 2.6 FoxO3-NETT@SMs restore H_2_O_2_ treated OA chondrocytes of mitochondrial dysfunction and inhibiting apoptosis

To assess the biocompatibility of FoxO3-NETT@SMs hydrogels, the upper chamber of *trans*-well inserts with 8 μm pores (Corning, USA) were filled with SMs, NETT@SMs and FoxO3-NETT@SMs, while the bottom 24-well plate of the Transwell inserts were seeded with IL-1*β* and 500 μmol/L H_2_O_2_-induced chondrocytes (1 × 10^4^ cells).

To observe the morphological changes of mitochondria using TEM, the following steps were performed: Chondrocytes were digested, and cells were collected by centrifugation at 1200×*g* for 6 min; The collected cells were fixed separately with 3% glutaraldehyde solution and 1% osmium tetroxide solution; The fixed cell samples were then dehydrated using acetone; After dehydration, the samples were infiltrated with Epon 812 resin for a prolonged period and then embedded; Methylene blue was used to stain semi-thin sections; Uranyl acetate and lead citrate were used to stain ultra-thin sections; Finally, the sections were observed using a transmission electron microscope JEM-1400-FLASH.

To assess mitochondrial membrane potential in chondrocytes, the JC-1 Mitochondrial Membrane Potential Detection Kit (Solarbio, Beijing, China) was used. The cells were processed and stained with JC-1 dye according to manufacturer's instructions, and observed using a fluorescence microscope (Olympus, Japan). Subsequently, in order to analyze stained cells, we used a BD Accuri C6 flow cytometer (BD Biosciences, USA) to measure the fluorescence intensity of both the red (FL2) and green (FL1) channels for each cell. The changes in mitochondrial membrane potential were evaluated based on the ratio of FL2 to FL1 signal intensity using ImageJ software.

The intracellular generation of ROS was detected using Cell Reactive Oxygen Species Detection Kits (Beyotime, China). Cells were treated with 10 μmol/L 2ʹ,7ʹ-dichlorofluorescin diacetate (DCFH-DA) for 30 min, followed by washing with PBS three times. After that, the cells were observed with a fluorescent microscope (Olympus, Japan). The semiquantitative analysis of fluorescence intensity using Image J software. The green fluorescence signals of individual cells were analyzed using a BD Accuri C6 flow cytometer (BD Biosciences, San Jose, CA, USA).

The apoptosis rate of chondrocytes was detected using the Annexin V-FITC/PI assay kit (4A Biotech). Following the instructions provided in the manual, chondrocytes were collected, washed, and then incubated with PI (20 mg/μL) and Annexin V-FITC dye for 5 min in the darkness. The apoptosis rate of the sample cells was determined using a BD Accuri C6 flow cytometer (BD Biosciences, San Jose, CA, USA).

### 2.7 Effects of FoxO3-NETT@SMs on IL-1β-induced chondrocytes *in vitro*

To assess the biocompatibility of FoxO3-NETT@SMs hydrogels microspheres, four groups of hydrogels microspheres, including SMs, NETT@SMs, and FoxO3-NETT@SMs, were placed in the upper chamber of the Transwell inserts (8 μm pores, Corning, USA). Simultaneously, IL-1*β*-induced chondrocytes (1 × 10^4^ cells) were seeded in 24-well plates for culture. CCK-8 assay (Dojindo, Japan) was used to assess cell proliferation after co-culturing OA chondrocytes with SMs, NETT@SMs, and FoxO3-NETT@SMs samples for 1, 3, and 7 days. Live/dead staining and phalloidin staining were performed for cell viability and morphology observation under CLSM, and the cell spreading area of cells co-culturing with SMs, NETT@SMs, and FoxO3-NETT@SMs samples was quantified using ImageJ software.

To investigate the effect of FoxO3-NETT@SMs hydrogel microspheres on cell migration ability, three groups of hydrogels microspheres, including SMs, NETT@SMs, and FoxO3-NETT@SMs, were placed in the bottom of a 24-well plate using Transwell inserts. Suspensions of IL-1*β*-induced chondrocytes (1 × 10^4^ cells) were added to the upper chamber of the Transwell inserts. After 24 h of culture using this setup, the cells on the upper side of the Transwell inserts were fixed, while those that migrated to the lower chamber were stained with crystal violet and observed under an optical microscope. Furthermore, the stained dye was dissolved in acetic acid solution, and the absorbance (OD) at 570 nm was measured to quantify the number of migrated cells in different groups. Additionally, scratch healing experiments were conducted by seeding 1 × 10^5^ IL-1*β*-induced chondrocytes co-culturing with SMs, NETT@SMs, and FoxO3-NETT@SMs samples in each well of a 12-well plate. The cell monolayer was scratched, and wound closure was assessed after 12 h of treatment with SMs, NETT@SMs, and FoxO3-NETT@SMs extracts.

Histological staining assays were conducted by fixing IL-1*β*-induced chondrocytes cultured with SMs, NETT@SMs, and FoxO3-NETT@SMs hydrogels for 3 and 7 days, followed by staining with toluidine blue and safranin O. Semi-quantification of staining results was performed using ImageJ software.

After co-culturing IL-1*β*-induced chondrocytes with SMs, NETT@SMs, and FoxO3-NETT@SMs hydrogels for 3 days and 7 days, cell samples were collected. Following the instructions provided in the respective kits, cells were processed for DNA quantification using the Picogreen DNA assay kit and for glycosaminoglycan (GAG) quantification using the Blyscan sulfated glycosaminoglycan assay kit. These analyses were conducted to determine the impact of different hydrogel groups on the secretion of extracellular matrix by OA chondrocytes.

Immunofluorescent staining assays were performed on fixed cells using SOX9 and COL2 antibodies, and the nucleus was stained with DAPI. Images were acquired using CLSM, and semi-quantitative analysis was performed using ImageJ software. mRNA expression was analyzed by incubating cells with SMs, NETT@SMs, and FoxO3-NETT@SMs hydrogels for 3 and 7 days, followed by total RNA extraction, reverse transcription, and RT-PCR. The relative mRNA expression levels of *Pink1*, *Col2*, *Col10*, *Adamts5*, *Sox9*, *IL-1β*, *Tnf-α*, *Foxo3*, *IL-10*, *Lc3b*, *Acan*, *P62*, and *Parkin* were determined using the comparative cycle threshold (CT) method (ΔΔCT method). All experiments were conducted in triplicate, and primer sequences are listed in Table S1.

### 2.8 DMM-induced OA rats

All animal experiments received approval from the Sichuan University Ethics Committee (Protocol Number KS2020330). Medial meniscus (DMM) surgery was performed on SD rats (male, 220–230 g) under sodium pentobarbital anesthesia. After anesthetizing the rats, the surgical area of the knee joint was exposed and sterilized. The joint cavity was then opened, and the anterior cruciate ligament was transected. Subsequently, microsurgical transection of the medial meniscus was performed, and the incision was sutured. In the Sham group, the joint capsule was incised without transection of the anterior cruciate ligament and medial meniscus to perform a sham operation. Four weeks after joint surgery, with the exception of the sham group (*n* = 20), the rats were divided into four groups (*n* = 20) in random order, which were injected intra-articularly with 100 μL of PBS, SMs, NETT@ SMs, and FoxO3-NETT@ SMs at weeks 5 and 6 post-surgery, respectively. In weeks 8 and 12 following surgery, the animals were sacrificed, and their knee joints were collected for analysis.

### 2.9 Gait analysis

The gait analysis for the rats at weeks 8 post-surgery was conducted using a system for analyzing the gait of animals (VisuGait, Shanghai, China). The footprints from walking spontaneously of rats were collected by placing each rat individually in the walkway, allowing them to walk freely. An analysis of gait changes was conducted using software. The recorded parameters included the stride length, walking speed, gait cycle, and average intensity.

### 2.10 Micro-CT analysis

After euthanizing the rats, the tissue fixation procedure was performed on their knee joints. In this study, joints were scanned with a micro-CT scanner (NMC-100, Pingsheng Medical Technology Co., Ltd., China) with high resolution (15 μm) at 90 kV (0.06 mA). Following the acquisition of imaging data, 3D reconstruction and bone density analysis were conducted by Recon 1.6.9.3 and Avatar 1.6.9.3 software. Statistics were applied to the data to determine the parameters that relate to subchondral bone, including trabecular bone volume fraction (Tb.BV/TV), trabecular number (Tb.N, mm^−1^), trabecular bone surface to bone volume ratio (Tb.BS/BV, mm^−1^), trabecular thickness (Tb.Th, mm), cortical bone mineral content (Ct.BMC, mg), and trabecular separation (Tb.Sp, mm).

### 2.11 Histological staining

After weeks 8 and 12 post-surgery, rats from different groups were euthanized, and knee joint specimens were obtained. The specimens were immersed in 4% paraformaldehyde solution (Sigma–Aldrich) for 2 days and then transferred to decalcification solution (10% ethylenediaminetetraacetic acid, Sigma–Aldrich) for 1 month. Subsequently, the decalcified knee joint tissue specimens were embedded in paraffin (Thermo Fisher Scientific) and sectioned into 6 μm-thick slices. The sections from different groups were stained with hematoxylin and eosin (H&E) and Safranin O-Fast Green (Sigma–Aldrich) to analyze and evaluate the joint tissues. Afterwards, based on the Safranin O-Fast Green staining results, the sections from different groups were graded according to the scoring criteria of the Osteoarthritis Research Society International (OARSI) for Safranin O-Fast Green staining and the thickness of the articular cartilage was calculated to systematically quantify the level of healthy cartilage.

### 2.12 Immunohistochemical and immunofluorescence staining

The paraffin sections mentioned above were further subjected to immunohistochemistry (IHC) and immunofluorescence (IF) staining. Firstly, the sections were deparaffinized, followed by antigen retrieval using standard methods. Subsequently, the sections were blocked with 1.5% goat serum and then incubated overnight at 4 °C with antibodies against COL2, COL10, AGG, PINK1, PARKIN, FOXO3, LC3B, and P62. For sections incubated with COL2, COL10, and AGG antibodies, DAB (3,3ʹ-diaminobenzidine, Vector Laboratories, Burlingame, CA, USA) was used for visualization according to the manufacturer's instructions, followed by counterstaining with hematoxylin for nuclear staining. For sections incubated with PINK1, PARKIN, FOXO3, LC3B, and P62 antibodies, immunofluorescence (IF) analysis was performed. These sections were incubated with secondary antibodies (labeled with Alexa 488 or Alexa 594 dyes) and counterstained with DAPI for nuclear staining. Immunohistochemistry slides were imaged under a bright-field microscope (Zeiss Axiovert 200, Oberkochen, Germany), while immunofluorescence slides were imaged under a fluorescence microscope (Zeiss Axiovert 200, Oberkochen, Germany). Finally, semi-quantitative analysis of positively stained cells for COL2, COL10, AGG, PINK1, PARKIN, FOXO3, LC3B, and P62 was performed using ImageJ software.

### 2.13 RT-PCR tests

A 12-week post-operative sample of joint cartilage was harvested for mRNA expression analysis. RT-PCR was employed to detect the mRNA expression of *Mmp13*, *Col2*, *Adamts5*, *Sox9*, *Tnf-α*, *Col10*, *Acan*, *IL-1β*, *Foxo3*, *Pink1*, *Parkin*, *P62* and *Lc3b*. Using the comparative cycle threshold (CT) method (ΔΔCT method), all experiments were performed in triplicate, and relative mRNA expression was calculated. Detailed primer information for different genes is provided in Supporting Information Table S1.

### 2.14 Transcriptome analysis

A transcriptome analysis performed by OE Biotech Co., Ltd. (Shanghai, China) evaluated FoxO3-NETT@SMs hydrogel functionality *in vivo* after 8 weeks. TRIzol reagent used for RNA extraction, a NanoDrop 2000 spectrophotometer and an Agilent 2100 bioanalyzer were used to estimate RNA purity and quantity. RNA-seq libraries were then prepared using the VAHTS Universal V6 RNA-seq Library Preparation kit. OE Biotech Co., Ltd. conducted sequencing and analysis of the transcriptome. Sequencing of the libraries was performed using the Illumina Novaseq 6000 platform, resulting in the generation of paired-end reads with a length of 150 bp. Each sample was generated as raw readings in fastq format. In order to obtain clean readings, raw readings were processed using fastp1 to remove low-quality data. HISAT22 was used to map clean readings from each sample to the reference genome. We calculated the FPKM3 of each gene. HTSeq-count4 was used to obtain the read counts of each gene. For differential expression analysis and bio-reproducibility assessment, we used DESeq25 and performed PCA analysis using R (v 3.2.0). For DEGs to be considered significantly differentially expressed, the *q*-value must be less than 0.05 and the fold change must be over 2.0.

### 2.15 Statistical analysis

The data are presented as mean ± standard deviation (S.D.). Three representative experiments were analyzed using OriginPro 2022 (OriginLab Corporation, USA) for means and standard deviations. Student's *t*-test, one-way or two-way ANOVA, followed by a Tukey post-hoc comparison was performed using GraphPad Prism software (GraphPad Software Inc.). Significance levels are denoted as follows: ∗*P* < 0.05, ∗∗*P* < 0.01, and ∗∗∗*P* < 0.001.

## 3 Results

### 3.1 FoxO3 downregulates the mitochondrial dysfunction associated with OA

**Figure 1:**
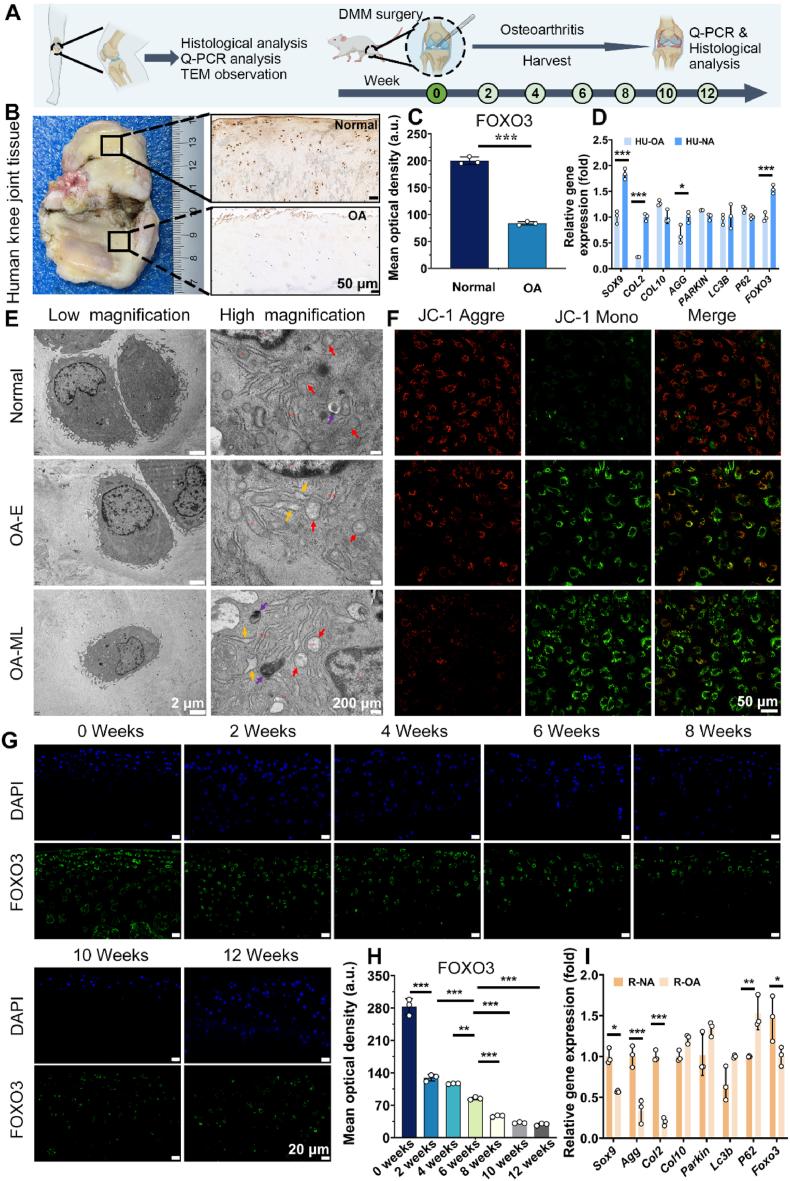
*Foxo3* downregulation in OA associated with mitochondrial dysfunction. (A) Schematic diagram of relevant studies performed on cartilage specimens from OA patients as well as articular specimens from OA rats at different stages of DMM modeling. (B) Representative images of gross observation and FOXO3 IHC staining in cartilage isolated from patients with knee OA, normal area (top), OA area (below); scale bar = 50 μm. (C) Semi-quantitative positive staining intensity of FOXO3 images. (D) Relative gene expression in cartilage tissue of patients with OA or normal was detected by qRT-PCR. (E) Representative TEM images of normal, early-stage osteoarthritic cartilage (OA-E), and middle- or late-stage osteoarthritic cartilage (OA-ML) isolated from patients with OA. (F) Representative JC-1 staining images of chondrocytes isolated from normal, early-stage osteoarthritic cartilage (OA-E), and middle- or late-stage osteoarthritic cartilage (OA-ML). (G) Representative images of FOXO3 IF staining in articular cartilage of rats euthanized at the indicated time points after DMM surgery; scale bar = 20 μm. (H) Semi-quantitative positive staining intensity of FOXO3 images. (I) Relative gene expression in cartilage tissue of rats euthanized at the indicated time points after DMM surgery was detected by qRT-PCR. *n* = 3. ∗*P* < 0.05, ∗∗*P* < 0.01, and ∗∗∗*P* < 0.001.

To explore *FOXO3*'s role in clinical OA and its impact on mitochondrial dynamics homeostasis, a series of biological evaluations on human joint tissues and rodent joint tissues were conducted (Fig. 1A). Human cartilage from healthy and osteoarthritic individuals was stained with immunohistochemistry (IHC) and examined using quantitative real-time RT-PCR analysis. IHC staining images of FOXO3, along with a semiquantitative analysis of its positivity indicated an obvious loss of FOXO3 protein expression in OA human cartilage (Fig. 1B and C). Similarly, qRT-PCR analysis of chondrocytes extracted from the above OA cartilage samples confirmed the reduction of *FOXO3* gene expression led to the downregulation of autophagy associated genes (*PARKIN*, *LC3B* and *P62*) and genes associated with cartilage matrix (*SOX9*, *COL2* and *AGG*), as well as the upregulation of hypertrophic marker *COL10* (Fig. 1D). In the examination of mitochondrial changes in OA tissues, transmission electron microscopy (TEM) images of normal and osteoarthritic human cartilage displayed distinct patterns (Fig. 1E). In the normal group (Normal), mitochondrial structures appeared typical with a mild autophagic response. In the mild osteoarthritis group (OA-E), mitochondria exhibited mild swelling, accompanied by slight expansion of the rough endoplasmic reticulum. In the moderate to severe osteoarthritis group (OA-ML), mitochondria exhibited significant swelling, and the rough endoplasmic reticulum displayed mild expansion, occurring simultaneously with autophagy. Assessment of mitochondrial membrane potential through dye JC-1 (5,5ʹ,6,6ʹ-tetrachloro-1,1ʹ,3,3ʹ-tetraethylbenzimi-dazolylcarbocyanine iodide) fluorescence staining revealed a heightened ratio of JC-1 aggregate to JC-1 monomer in the normal group, indicating a healthy mitochondrial functional state^38^^,^^39^. Conversely, in the E-OA and ML-OA groups, a substantial reduction in the ratio of JC-1 aggregate to JC-1 monomer was identified, indicating mitochondrial dysfunction in osteoarthritis tissues (Fig. 1F and Supporting Information Fig. S3).

The aforementioned analysis was also performed on knee cartilage from Sprague–Dawley rats who had either undergone sham surgery or the destabilization of the medial meniscus (DMM) surgery to induce post-traumatic osteoarthritis (PTOA), mimicking the clinically pathological development and progression of OA. As shown in Fig. 1G and H, FoxO3 protein expression gradually silenced in a time-dependent manner as OA progressed to late stages in cartilage tissue. Moreover, *FOXO3* gene expression in chondrocytes decreased following the same trend as other cartilage-specific genes, such as *SOX9*, *AGG*, *COL10* and *COL2* and autophagy associated genes, such as *PARKIN*, *LC3B* and *P62* (Fig. 1I). Collectively, these results suggest the low expression of *FOXO3* is observed in OA tissues, which is accompanied by mitochondrial dysfunction.

### 3.2 Fabrication and characterization of FoxO3-NETT

**Figure 2:**
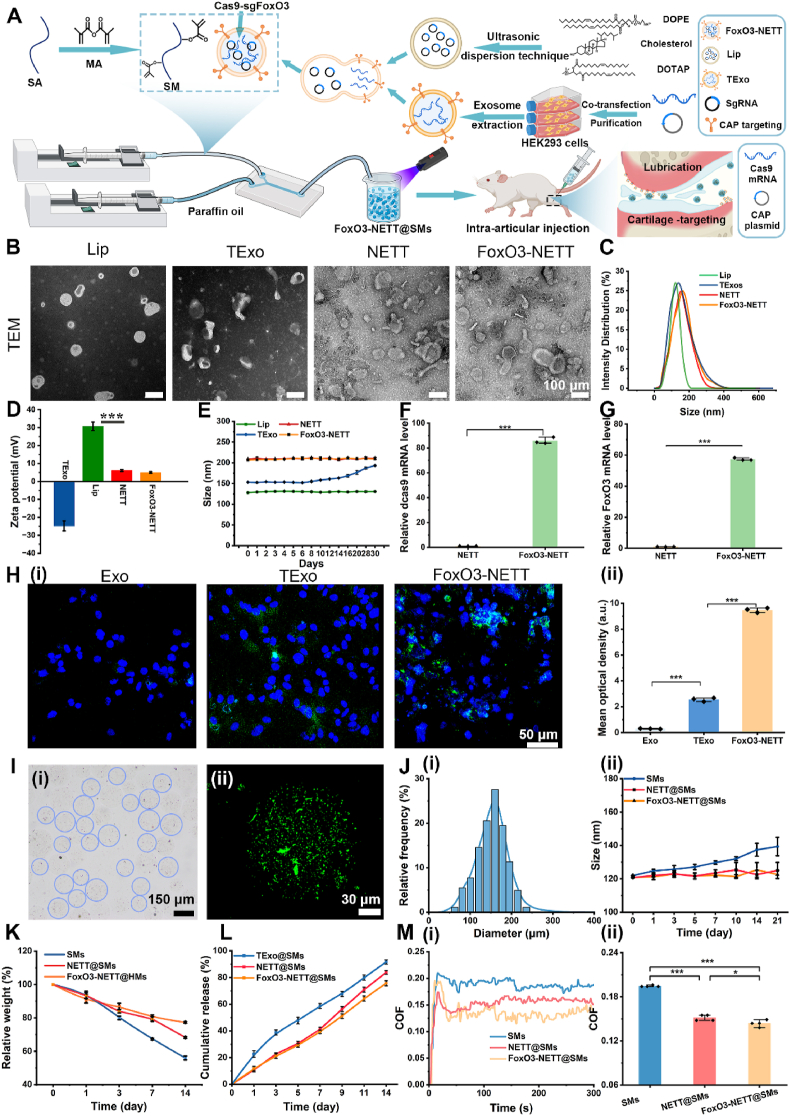
Fabrication and characterization of FoxO3-NETT@SMs. (A) The flow diagram for the preparation of FoxO3-NETT@SMs. (B) TEM images of Lip, TExo, NETT and FoxO3-NETT. scale bar = 100 μm. (C) The size distribution of Lip, TExo, NETT and FoxO3-NETT. (D) The surface zeta potential of Lip, TExo, NETT and FoxO3-NETT. (E) The particle size stability of Lip, TExo, NETT and FoxO3-NETT at the indicated time points in PBS solution. (F) The dCas9 mRNA expressions in NETT and FoxO3-NETT was detected by qRT-PCR. (G) The *Foxo3* expression levels of rat chondrocytes after incubating with NETT and FoxO3-NETT after 24 h *in vitro*. (H(i)) Representative fluorescence images of DIO-labeled Exo, TExo and FoxO3-NETT (stained with green fluorescence) after incubated with chondrocytes (nuclei stained with blue color) for 4 h *in vitro*. Scale bar = 50 μm. (H(ii)) Semi-quantitative green fluorescence staining intensity of images in H(i). (I(i)) Bright-field images of transparent FoxO3-NETT@SMs. (I(ii)) Representative CLSM images of FoxO3-NETT@SMs (DIO-labeled FoxO3-NETT stained with green fluorescence). (J(i)) Size distribution of FoxO3-NETT@SMs. (J(ii)) Size stability of SMs, NETT@SMs and FoxO3-NETT@SMs at the indicated time points in PBS solution. (K) The degradation curve of SMs, NETT@SMs and FoxO3-NETT@SMs. (L) Release curves of protein-containing vesicles releasing from TExo@SMs, NETT@SMs and FoxO3-NETT@SMs. (M(i)) COF-time curves and (M(ii)) COF histograms for SMs, NETT@SMs and FoxO3-NETT@SMs. *n* = 3, ∗*P* < 0.05 and ∗∗∗*P* < 0.001.

The fabrication process of FoxO3-NETT is demonstrated in Fig. 2A. The “Cargo” (Cas9-sgFoxO3), which regulates autophagic activation, was designed and fabricated by a CRISPR/Cas9-based FoxO3-targeted gene-editing tool (Figs. S1 and S2). To ensure specific delivery of the “Cargo” (Cas9-sgFoxO3) to OA chondrocytes for ameliorating mitochondrial dysfunction, a chondrocyte-targeting nanoengineered “truck” (NETT) loaded with “Cargo” was developed. To achieve this, liposomes containing SgFoxO3 plasmids (Lip) were fused to exosomes containing Cas9 protein (TExo, Figs. S1 and S2) to target chondrocytes, which is referred as FoxO3-NETT. For comparison, NETT was fabricated as a control using the same process, but without loading the FoxO3 gene-editing tool. TEM images confirmed the nano-sized spherical vesicle morphology of Lip, TExo, NETT, and FoxO3-NETT (Fig. 2B). As illustrated in Fig. 2C, the average sizes of Lip, TExo, NETT, and FoxO3-NETT were 125.39 nm, 139.82 nm, 153.69 nm, and 163.44 nm, respectively. The surface charges of Lip, TExo, NETT, and FoxO3-NETT were further assessed by DLS. Despite the negative charge of −24.78 ± 2.82 mV in TExo and a positive charge of 31.10 ± 2.20 mV in Lip, there was a slight positive surface zeta potential for NETT as well as FoxO3-NETT (Fig. 2D). The mild positive charge on NETT and FoxO3-NETT particles may help them target more efficiently inside joints. This is attributed to the subtle positive charge on their surfaces, which allows them to adhere better to negatively charged surfaces of chondrocytes and glycosaminoglycan chains within cartilage tissue^40^. The stability of the delivery vesicle is crucial for OA therapy. It can be seen from Fig. 2E that the particle size of both NETT and FoxO3-NETT was consistently constant after 30 days, whereas TExo gradually increased after Day 6. Additionally, RT-PCR testing was employed to assess the loading efficiency and effectiveness of the CRISPR/Cas9-based FoxO3-targeted gene-editing tool (Cas9-sg FoxO3) in FoxO3-NETT, confirming successful loading of Cas9-sg FoxO3 in FoxO3-NETT (Fig. 2F and Supporting Information Fig. S4). Furthermore, Fig. 2G demonstrated that FoxO3-NETT promotes *Foxo3* gene expression in chondrocytes by delivering the gene editor Cas9-sgFoxO3. To verify the enhanced uptake efficiency of FoxO3-NETT in chondrocytes *in vitro*, DIO-labeled natural EXO, TExo, or FoxO3-NETT were incubated with chondrocytes for 4 h. The FoxO3-NETT group showed higher green fluorescence signals (blue nucleus) than either EXO or TExo groups, suggesting more FoxO3-NETT were taken up by chondrocytes (Fig. 2H(i)). The enhanced uptake of FoxO3-NETT within chondrocytes is attributed to the weak positive surface charge, which is more favorable to bind the negatively charged chondrocyte surface *via* electrostatic interaction, while the strong negative charge of TExo results in the electrostatic repulsion between the nanoparticles and cells^41^. Collectively, these results confirm the enhanced chondrocyte-targeting of FoxO3-NETT. Semi-quantitative analysis of mean optical density (Fig. 2H(ii)) further consolidated previous conclusions. Based on these findings, FoxO3-NETT is proven to be effective in chondrocyte-specific targeting *in vitro*.

### 3.3 Fabrication and characterization of injectable hydrogel microspheres FoxO3-NETT@SMs

To ensure stable and sustained release of FoxO3-NETT within the joint cavity, microfluidic technology was utilized to encapsulate FoxO3-NETT in sodium alginate hydrogel microspheres. The fabrication process of these injectable hydrogel microspheres (FoxO3-NETT@SMs) is depicted in Fig. 2A. Sodium alginate (SA) was modified with methacrylic anhydride (SMs) to integrate photocuring properties. Methacrylate groups have been successfully grafted based on proton nuclear magnetic resonance (^1^H NMR) spectra (Supporting Information Fig. S5). A microfluidic device was employed to generate pre-gel droplets containing either NETT or FoxO3-NETT and SMs. These droplets were subsequently photo-polymerized to form three types of hydrogel microspheres: SMs, NETT@SMs, and FoxO3-NETT@SMs. Optical microscopy images revealed that these hydrogel microspheres exhibited a uniform size distribution and maintained excellent morphological integrity (Fig. 2I(i)). To confirm the successful incorporation of FoxO3-NETT into SMs, confocal laser scanning microscopy (CLSM) was used to detect Dio-labeled FoxO3-NETT within the SMs. As depicted in Fig. 2I(ii), green fluorescence was evenly distributed inside the SMs, indicating successful encapsulation of FoxO3-NETT within the SMs. The average particle size of hydrogel microspheres was determined to be 160.5 ± 6.49 μm, with a narrow size distribution (Fig. 2J(i)). This size is anticipated to facilitate the injection and lubricated movement of the hydrogel microspheres within the joint cavity, reducing the wear and tear on knee joints. As shown in Fig. 2J(ii), in PBS solution, the NETT and FoxO3-NETT@SMs groups demonstrated higher size stability compared to SMs alone. Specifically, the size of NETT and FoxO3-NETT@SMs remained unchanged for at least 21 days, while the size of SMs gradually increased.

Degradation experiments in PBS for SMs, NETT@SMs, and FoxO3-NETT@SMs yielded similar results (Fig. 2K). The degradation rate of SMs was significantly faster than of FoxO3-NETT@SMs, with SMs degrading approximately 44% on the 14th day, while FoxO3-NETT@SMs degraded only about 23%. In drug release experiments, the cumulative release rate of protein drugs from FoxO3-NETT@SMs was significantly slower than that from TExo@SMs. On the 14th day, TExo@SMs released approximately 90%, while FoxO3-NETT@SMs released only about 75%, indicating a higher degree of controlled drug release for FoxO3-NETT@SMs (Fig. 2L). The differing release rates between FoxO3-NETT@SMs and TExo@SMs may be attributed to the covalent molecular forces between the cationic FoxO3-NETT and entangled anionic sodium alginate polymer chains, resulting in a tighter network structure, thereby achieving slow and controlled release of FoxO3-NETT. In conclusion, these results indicate that FoxO3-NETT@SMs effectively protect FoxO3-NETT, allowing for sustained release within the joint cavity.

To evaluate the lubrication performance of freshly prepared FoxO3-NETT@SMs, NETT@SMs, and SMs, friction tests lasting 800 s were conducted on a high-frequency reciprocating friction and wear tester (Supporting Information Fig. S6). As illustrated in Fig. 2M(i), the friction curves for FoxO3-NETT@SMs, NETT@SMs, and SMs remain relatively stable over time. Notably, the coefficient of friction (COF) for FoxO3-NETT@SMs exhibits a slight decrease around the 100-s mark, remaining at a lower COF level afterward. This improvement in lubrication can be attributed to the lipid-based boundary mechanism. As a result, the COF values for SMs were significantly higher than those for NETT@SMs and FoxO3-NETT@SMs (Fig. 2M(ii)). These findings confirm the superior lubrication performance of FoxO3-NETT@SMs. The intra-articular injection of FoxO3-NETT@SMs provides considerable benefits, including reduced friction, improved lubrication, and prevention of joint wear and tear within the joint cavity.

### 3.4 FoxO3-NETT@SMs restore mitochondrial function and inhibit apoptosis in H_2_O_2_-treated OA chondrocytes

**Figure 3:**
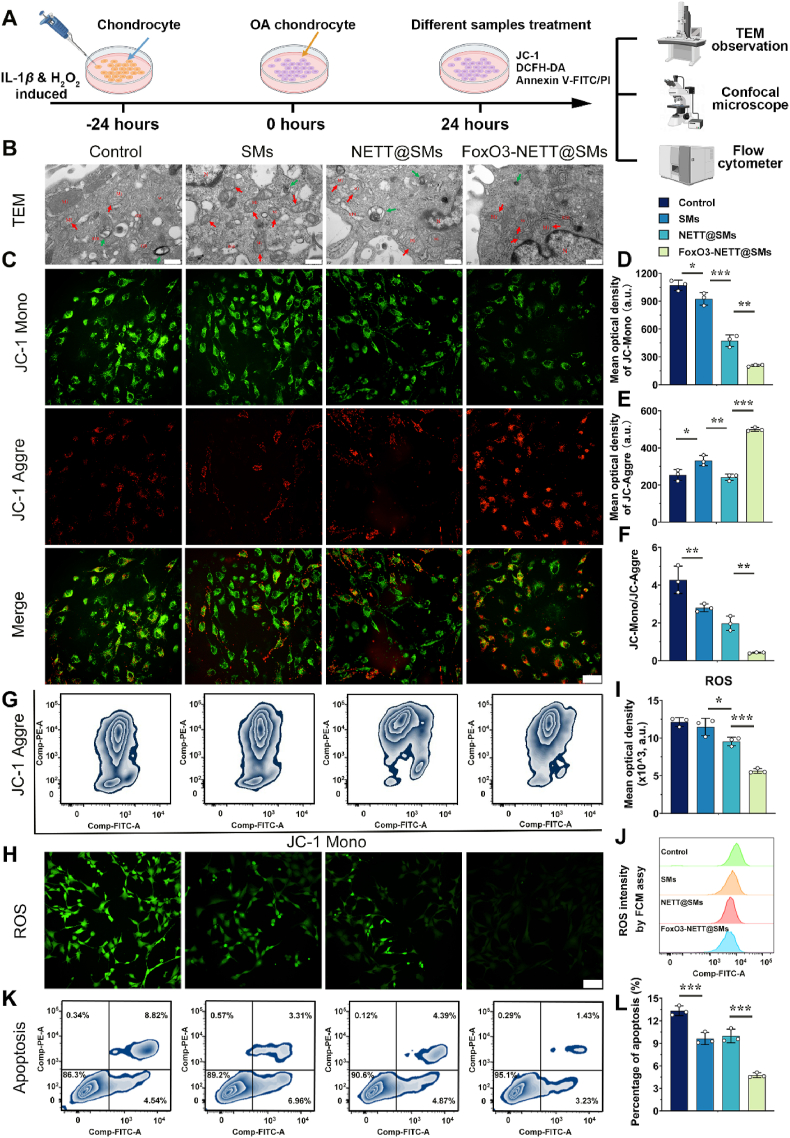
The ability of inhibiting apoptosis and restoring mitochondrial dysfunction of FoxO3-NETT@SMs *in vitro*. (A) The schematic diagram for the therapeutic effects of inhibiting apoptosis and restoring mitochondrial dysfunction of FoxO3-NETT@SMs on OA chondrocytes. (B) TEM images of H_2_O_2_ treated OA chondrocytes (Control), and H_2_O_2_ treated OA chondrocytes cultured with SMs, NETT@SMs and FoxO3-NETT@SMs. Scale bar = 500 nm. (C–F) Representative JC-1 staining images of OA chondrocytes after different drug treatments and to perform a semi-quantitative analysis of ratio of JC-Mono to JC-Aggre levels. Scale bar = 100 μm. (G) Mitochondrial membrane potential within OA chondrocytes after different drug treatments was detected by flow cytometry. (H, I) CLSM images of OA chondrocytes cultured with the ROS probe DCFH-DA (green) to directly observe intracellular ROS levels and to perform a semi-quantitative analysis of ROS levels. Scale bar = 100 μm. (J) Quantitative analysis of intracellular ROS levels in OA chondrocyte using FCM. (K, L) Quantitative analysis of OA chondrocyte apoptosis using FCM. *n* = 3, ∗*P* < 0.05, ∗∗*P* < 0.01, and ∗∗∗*P* < 0.001.

The accumulation of reactive oxygen species (ROS) triggers oxidative stress, leading to mitochondrial damage and dysfunction^42^. This cascade ultimately results in chondrocyte damage, senescence, and apoptosis, contributing to the progressive development of OA^43^^,^^44^. Therefore, restoring mitochondrial function and dynamic balance to prevent chondrocyte apoptosis and matrix degradation is crucial for effective OA treatment. An evaluation was conducted on the influence of various treatment groups on restoring mitochondrial function, dynamic balance, and apoptosis inhibition in OA chondrocytes under oxidative stress conditions (Fig. 3A). Three experimental groups were established, each receiving treatment with FoxO3-NETT@SMs, NETT@SMs, and SMs respectively, after the induction of chondrocytes with IL-1*β* and H_2_O_2_ to induce an oxidative stress state. The negative control group consisted of IL-1*β* and H_2_O_2_-induced chondrocytes.

TEM analysis revealed normal mitochondrial morphology in the FoxO3-NETT@SMs and blank groups (Fig. 3B and Supporting Information Fig. S7). Conversely, the control and SMs groups displayed notable mitochondrial swelling, mild expansion of the rough endoplasmic reticulum, and substantial inhibition of mitochondrial autophagy. Additionally, JC-1 was employed to assess mitochondrial membrane potential (MMP), revealing early apoptosis in chondrocytes under H_2_O_2_-induced damage. JC-1 aggregates (red fluorescence) and monomers (green fluorescence) represented normal MMP, early apoptosis and mitochondrial depolarization, respectively. Chondrocytes treated with FoxO3-NETT@SMs displayed MMP levels similar to the blank group, with a high JC-1 aggregate to JC-1 monomer ratio. In contrast, in the control and SMs groups, the fluorescence intensity of JC-1 aggregates was low while that of JC-1 monomers was high, indicating abnormal mitochondrial function and apoptotic status (Fig. 3C–G). Treatment with FoxO3-NETT@SMs effectively protected mitochondrial membranes, significantly suppressing the decline in MMP (Fig. 3C–G). Using the DCFH-DA probe, ROS levels inside cells were determined. As demonstrated in Fig. 3H, chondrocytes after treatment (control and SMs groups) displayed significantly increased intracellular ROS levels, which were markedly reduced with the addition of FoxO3-NETT@SMs (Fig. 3H), aligning with quantitative findings (Fig. 3I and J). Additionally, the double staining of Annexin V and Propidium iodide (PI) revealed a substantial reduction in apoptotic cells after FoxO3-NETT@SMs treatment (Fig. 3K and L). Therefore, based on the comprehensive data analysis, it is confirmed that FoxO3-NETT@SMs effectively ameliorate chondrocytes under oxidative stress by mitigating mitochondrial dysfunction and inhibiting apoptosis. This suggests the potential of FoxO3-NETT@SMs in reactivating mitochondrial autophagy, suppressing mitochondrial apoptosis, and restoring mitochondrial dysfunction and dynamic stability in the osteoarthritic environment.

### 3.5 FoxO3-NETT@SMs enhance proliferation, migration, and ECM synthesis of OA chondrocytes *in vitro*

**Figure 4:**
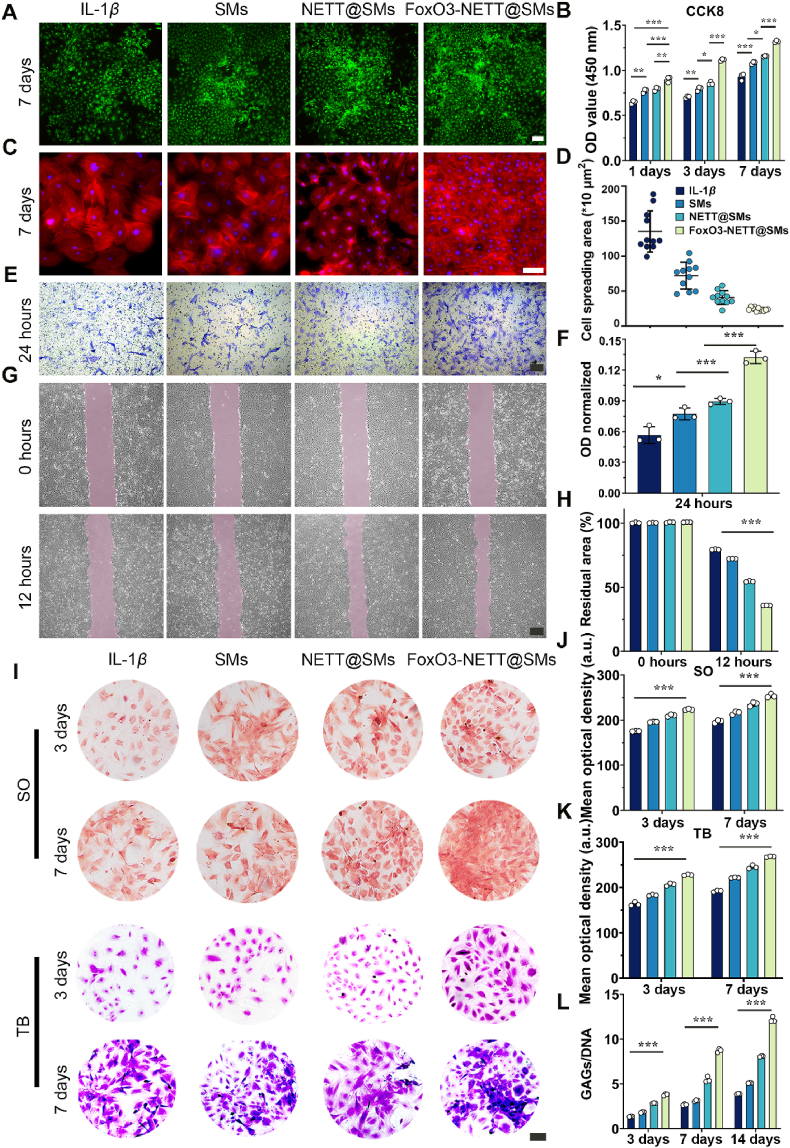
FoxO3-NETT@SMs promote proliferation and migration, and ECM synthesis of OA chondrocytes *in vitro*. (A) OA chondrocytes vitality incubated with SMs, NETT@SMs and FoxO3-NETT@SMs after 7 days measured by Live/Dead assay. Scale bar = 200 μm. (B) Proliferative activity of SMs, NETT@SMs and FoxO3-NETT@SMs on chondrocytes examined with CCK-8 assay. (C) OA chondrocytes cytoskeleton incubated with SMs, NETT@SMs and FoxO3-NETT@SMs after 7 days measured by Phalloidin staining, Scale bar = 100 μm. (D) The quantitation of cell spreading area according to the phalloidin staining images. (E) Effects of different samples on the migration of OA chondrocytes *in vitro*, Scale bar = 100 μm. (F) In migration experiment, the quantitative OD value of acetic acid dissolved crystal violet in each group at 570 nm. (G) Representative images showing the migration ability of OA chondrocytes cultured with different samples at 12 h by scratch wound assay. Scale bar = 500 μm. (H) Quantitative analysis of the migration residual area of scratch wound assay in each group. (I) Representative safranine O and TB staining images of OA chondrocytes incubated with different samples after 3 and 7 days. Scale bar = 50 μm. (J) Semi-quantitative analysis of safranine O staining in each group. (K) Semi-quantitative analysis of TB staining in each group. (L) Quantitative analysis of secretion of glycosaminoglycans levels in OA chondrocyte using GAG/DNA assay. *n* = 3, ∗*P* < 0.05, ∗∗*P* < 0.01, and ∗∗∗*P* < 0.001.

An IL-1*β*-induced osteoarthritis chondrocyte model was created to study the therapeutic effects of FoxO3-NETT@SMs on osteoarthritis chondrocytes. The induced chondrocytes were co-cultured with SMs, NETT@SMs, and FoxO3-NETT@SMs. The negative control group consisted of IL-1*β*-induced chondrocytes, referred to as the IL-1*β* group. Live/dead cell staining revealed that, as of day 7, FoxO3-NETT@SMs exhibited stronger cell viability compared to other groups (Fig. 4A and Supporting Information Fig. S8), indicating that FoxO3-NETT@SMs promote cell survival and inhibit apoptosis. CCK-8 experiments demonstrated a significant increase in cell proliferation activity following treatment with FoxO3-NETT@SMs (Fig. 4B). Phalloidin staining revealed improvements in the morphology of OA chondrocytes after co-culture with FoxO3-NETT@SMs (Fig. 4C and D, and Supporting Information Fig. S9), as evidenced by an enhanced elliptical shape and reduced spreading area. In the Transwell migration assay, the FoxO3-NETT@SMs group exhibited the highest migration of OA chondrocytes to the lower chamber, while the SMs and NETT@SMs groups presented reduced number of migrated OA chondrocytes (Fig. 4E). Quantitative OD values of crystal violet dissolution at 570 nm further verified these observations (Fig. 4F). Additionally, a cell migration scratch healing experiment was conducted. Fig. 4G illustrated that the migration area of the FoxO3-NETT@SMs group was significantly larger than the other groups. Concurrently, the residual quantitative area of wound healing in the FoxO3-NETT@SMs group was the smallest, signifying that FoxO3-NETT@SMs enhanced chondrocyte migration ability (Fig. 4H).

**Figure 5:**
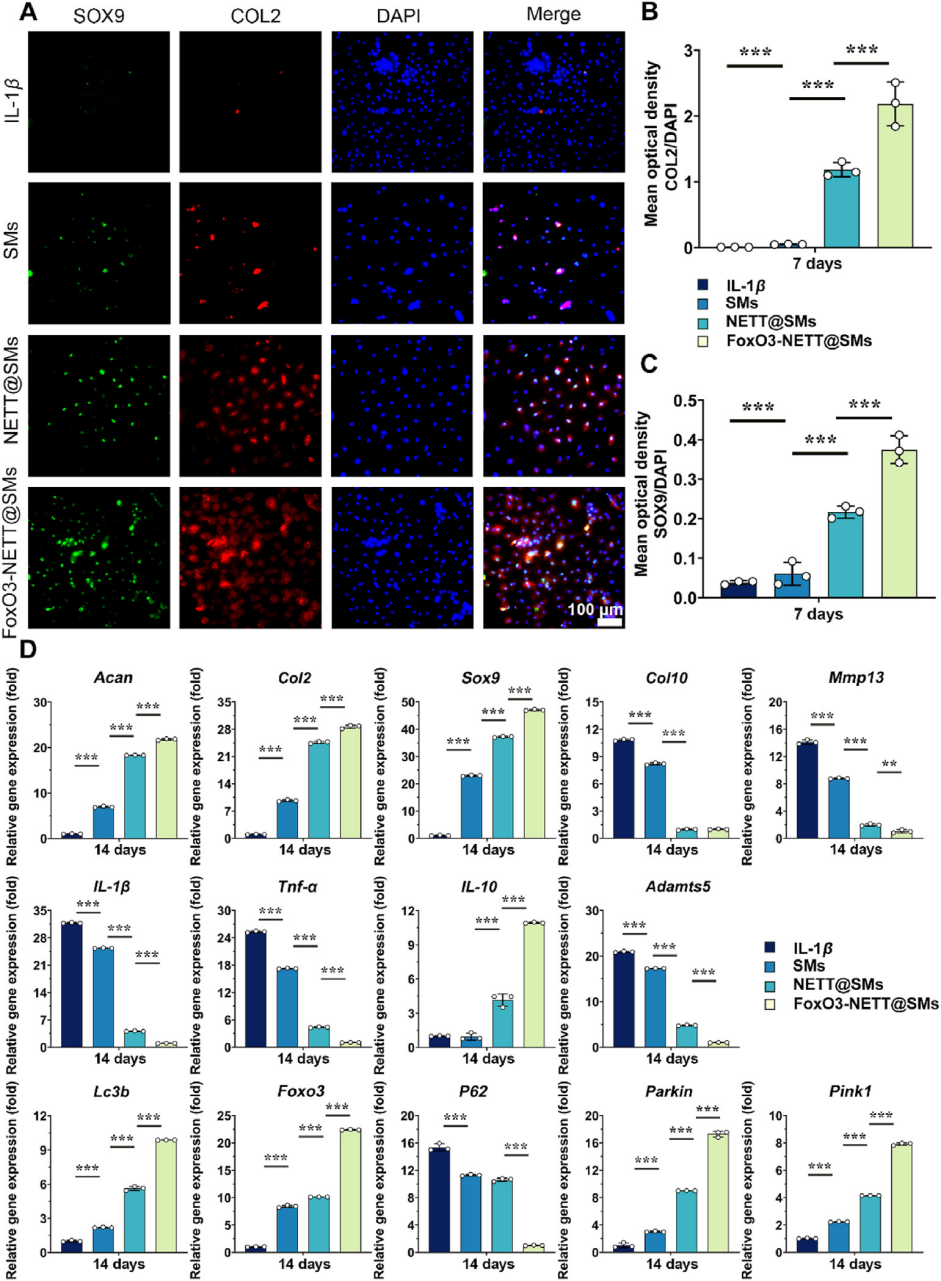
FoxO3-NETT@SMs accelerate ECM synthesis and cartilage matrix regeneration in OA chondrocytes *in vitro*. (A) Representative immunofluorescence staining images of cartilage matrix relative protein Col2 and Sox9 in OA chondrocytes incubated with different samples after 7 days, scale bar = 100 μm. (B) Semi-quantitative analysis of Col2 IF staining. (C) Semi-quantitative analysis of Sox9 IF staining. (D) The expression of cartilage-specific matrix genes, inflammatory relative genes autophagy associated genes and assessed by RT-qPCR assay at indicted time points. *n* = 3, ∗∗*P* < 0.01, and ∗∗∗*P* < 0.001.

To assess the effects of FoxO3-NETT@SMs on production of extracellular matrix components in OA chondrocytes, Safranin O and TB staining were performed on days 3 and 7 after different treatments. As shown in Fig. 4I, staining results of matrix associated with cartilage indicated a trend of FoxO3-NETT@SMs > NETT@SMs > SMs > OA group. Semi-quantitative staining results also mirrored this trend (Fig. 4J and K), suggesting that FoxO3-NETT@SMs can alleviate the protein polysaccharide loss in OA chondrocytes. Similarly, each sample is quantified for DNA and glycosaminoglycans (GAG) content, which showed a significant increase in polysaccharides that are specific to cartilage in FoxO3-NETT@SMs group (Fig. 4L and Supporting Information Fig. S10). Specific proteins’ (SOX9 and COL2) immunofluorescence staining was performed to examine how FoxO3-NETT@SMs affect regenerated cartilage matrix secreted by chondrocytes. As illustrated in Fig. 5A, under FoxO3-NETT@SMs treatment, the expression of both proteins was significantly enhanced. Fluorescence density-based semi-quantitative analysis (Fig. 5B and C) corroborated this trend. Moreover, RT-qPCR results in Fig. 5D also revealed that, compared to NETT@SMs, SMs and IL-1*β* controls, in OA chondrocytes treated with FoxO3-NETT@SMs, the expression levels of several matrix genes associated with cartilage, including *Sox9*, *Col2*, and *Agg* were significantly increased, while the expression levels of cartilage matrix degradation associated genes, including *Mmp13*, *Col10*, and *Adamts5*, were significantly decreased. Additionally, it was observed that with the significantly increased expression of the *Foxo3* gene in samples treated with FoxO3-NETT@SMs, *IL-1β* and *Tnf-α*, which are genes that promote inflammation, were decreased in expression, whereas the gene *IL-10*, which has anti-inflammatory properties, was increased, along with the upregulation of autophagy-related genes (*Lc3b*, *P62*, *Parkin* and *Pink1*). To this end, FoxO3-NETT@SMs can effectively promote the proliferation and migration of OA chondrocytes *in vitro*, enhance synthesis and quality of ECM, play a role in anti-inflammatory effect, enhance mitochondrial autophagy, and prevent further development of OA.

### 3.6 FoxO3-NETT@SMs effectively ameliorate progressive OA *in vivo*

**Figure 6:**
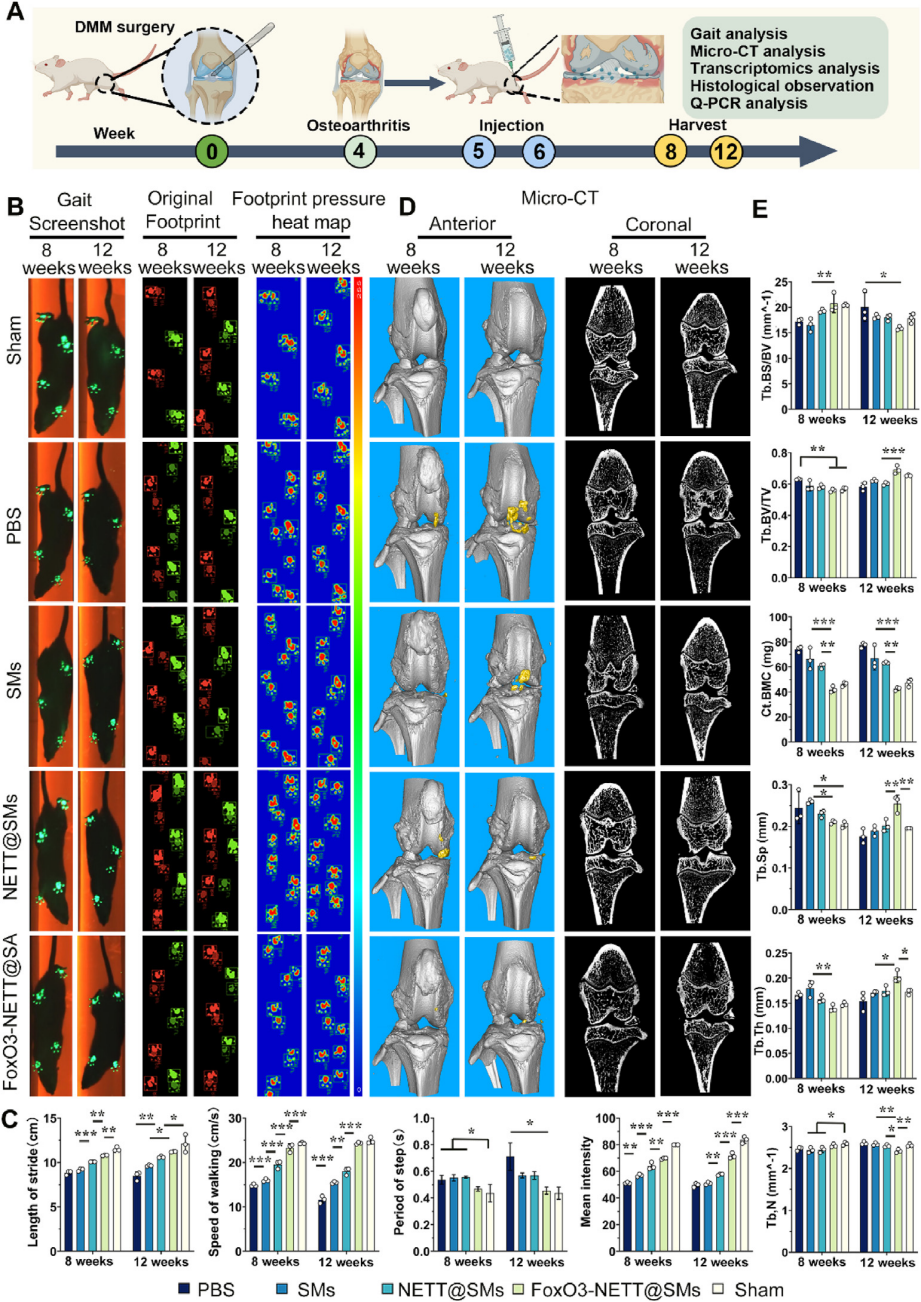
FoxO3-NETT@SMs effectively alleviate OA progression *in vivo*. (A) Schematic illustration of alleviating OA progression *in vivo*. (B) Representative gait screenshot, representative original footprint images, and representative footprint pressure heatmap images. (C) Quantification analysis of length of stride, speed of walking, period of steep and mean intensity. (D) Representative Micro CT images after 8- and 12-weeks joint cavity injection. (E) Quantification analysis of bone morphological parameters after 8- and 12-weeks joint cavity injection. *n* = 3, ∗*P* < 0.05, ∗∗*P* < 0.01, and ∗∗∗*P* < 0.001.

To evaluate FoxO3-NETT@SMs’ therapeutic effects on progressive cartilage loss and damage of OA cartilage, a rat OA model was established through DMM surgery (Fig. 6A). Positive controls consisted of normal rats who were not subjected to DMM surgery, while negative controls comprised DMM rats who were injected with PBS. To investigate the synergistic effects of the FoxO3-NETT@SMs system, three experimental groups were included: FoxO3-NETT@SMs, NETT@SMs, and SMs. Specifically, the SMs demonstrate the effect of SA hydrogel microspheres, the NETT@SMs indicate the combined effects of SA microspheres and enhanced lubrication attributed to the lipid-based boundary *via* the integration of NETT and SMs, and the FoxO3-NETT@SMs illustrate the collective effects of the system. Consequently, the individual effects of chondrocyte-targeted *Foxo3* gene-editing, the influence of SMs, and the enhanced lubrication can be respectively indicated by comparing the FoxO3-NETT@SMs *versus* NETT@SMs, SMs *versus* PBS, and NETT@SMs *versus* SMs groups.

After an 8- and 12-week postoperative period, gait analysis was conducted on rats subjected to intra-articular gel injection to evaluate knee joint movement, including gait index parameters such as gait snapshots, gait patterns, and gait intensity (Fig. 6B and C). No significant differences were observed in gait snapshots among the groups. However, the PBS, SMs, and NETT@SMs groups displayed abnormal gait patterns, characterized by irregular and asymmetrical steps, compared to the sham and FoxO3-NETT@SMs groups (Fig. 6B and Supporting Information Fig. S11). Moreover, independent analysis of the right hind limb parameters, including stride length, walking speed, step frequency, and average intensity, revealed closer similarities between the sham and FoxO3-NETT@SMs groups (Fig. 6C). The FoxO3-NETT@SMs group exhibited trends consistent with the sham group, indicating normal joint activity with larger stride, faster walking speed, shorter gait cycle, and higher average gait intensity compared to the PBS, SMs, and NETT@SMs groups.

At 8- and 12-weeks post-surgery, micro-CT imaging was conducted to assess the impact of different treatments on bone destruction in rats (Fig. 6D). Reconstructed CT images displayed the extent of bone damage and osteophyte formation in each treatment group (Fig. 6D and Supporting Information Fig. S12). 3D micro-CT image analysis revealed varying degrees of bone erosion and osteophyte formation in the knee joints of all treatment groups compared to the sham group. Notably, the FoxO3-NETT@SMs treatment group significantly prevented bone damage, loss of bone mass, and osteophyte formation. Bone morphometric parameter analysis suggested that various bone morphometric parameters in the FoxO3-NETT@SMs treatment group closely resembled those in the sham group (Fig. 6E). Subchondral bone-specific parameters, including the trabecular bone surface to bone volume ratio (Tb.BS/BV, mm^−1^), trabecular bone volume fraction (Tb.BV/TV), cortical bone mineral content (Ct.BMC, mg) and trabecular number (Tb.N, mm^−1^) in the FoxO3-NETT@SMs group showed no significant difference from the sham group. Over time, the 12-week 3D micro-CT images of the FoxO3-NETT@SMs treatment group exhibited fewer osteophyte formations compared to the 8-week images. The bone morphometric parameters were also closer to those of the sham group. Conversely, the severity of OA in the SMs and NETT@SMs groups worsened further over time, suggesting that FoxO3-NETT@SMs effectively prevented OA progression. In summary, the FoxO3-NETT@SMs treatment group demonstrated efficacy in preventing bone damage and erosion during the progression of OA, showcasing promising therapeutic effects.

**Figure 7:**
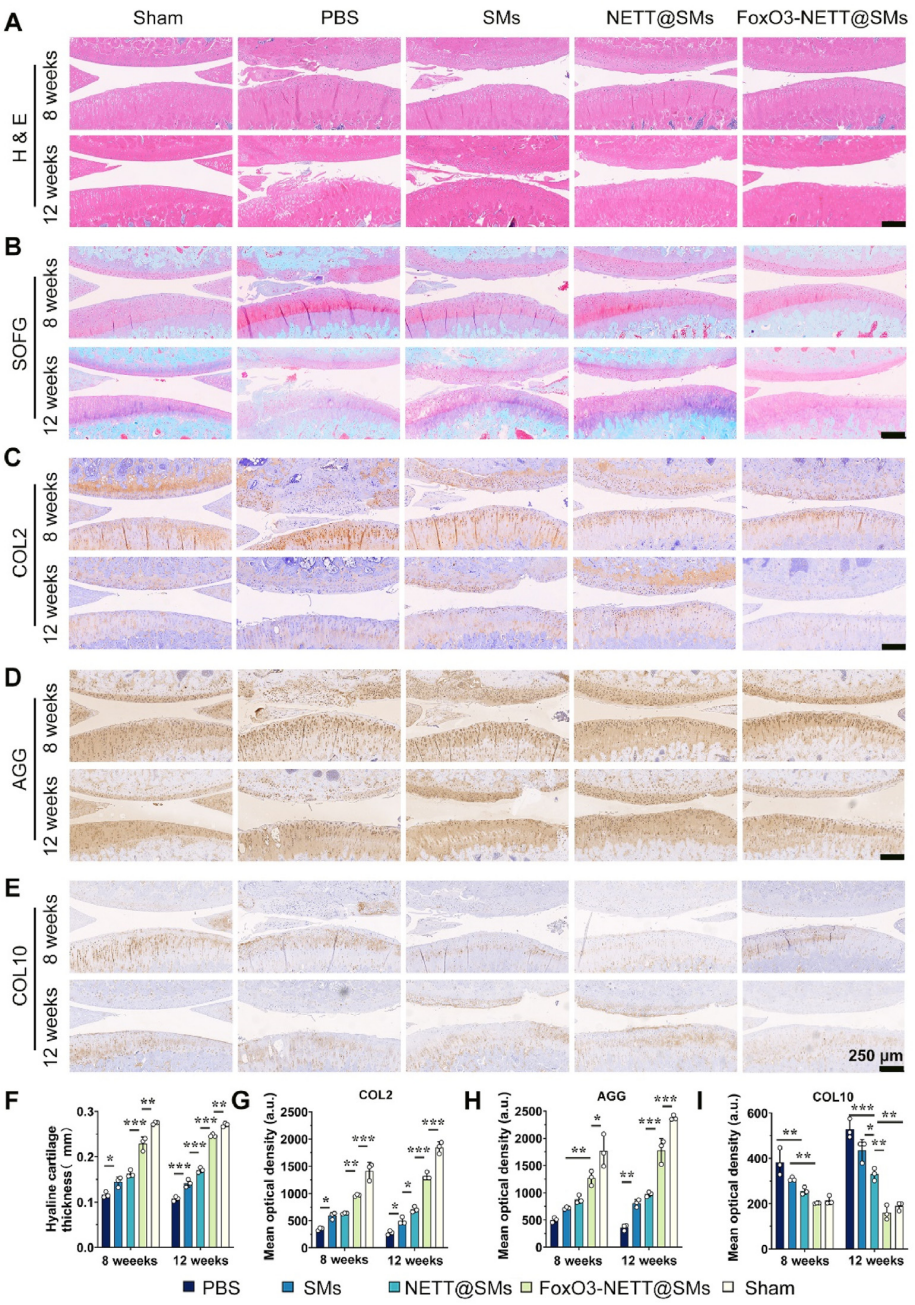
FoxO3-NETT@SMs effectively alleviate OA progression *in vivo*. Representative images of (A) HE staining and (B) Safranin O-fast green staining from each group after 8- and 12-weeks therapy. Scale bar = 250 μm. Representative IHC staining images of (C) COL2, (D) AGG and (E) COL10 from each group after 8- and 12-weeks therapy. Scale bar = 250 μm. (F) Quantification analysis of hyaline cartilage thickness according to Safranin O-fast green-stained sections. (G) Semi-quantitative analysis of COL2 IHC staining (H) Semi-quantitative analysis of AGG IHC staining. (I) Semi-quantitative analysis of COL10 IHC staining. *n* = 3, ∗*P* < 0.05, ∗∗*P* < 0.01, and ∗∗∗*P* < 0.001.

Given that cartilage tissue undergoes varying degrees of pathological damage during OA progression, the health of the cartilage layer emerges as a crucial indicator for assessing the degree of OA. After 8- and 12-weeks post-surgery, standardized processing of knee joint samples was performed, followed by H&E staining and Safranin O-Fast Green staining to assess the extent of cartilage tissue damage and examine the impact of FoxO3-NETT@SMs hydrogel on cartilage (Fig. 7A and B). The thickness and smoothness of cartilage indicated the degree of cartilage degradation, with thicker and smoother cartilage implying milder degradation in OA. In the OA model, uniformity in cartilage tissue staining was also necessary for determining cartilage degradation. The more uniform the cartilage staining, the less degradation of the cartilage. From the histological staining results, the H&E staining in the PBS group revealed a rough cartilage surface with uneven staining. In contrast, the FoxO3-NETT@SMs hydrogel group exhibited the most uniform staining, with a smoother cartilage surface, indicating minimal cartilage degradation. The uniformity of staining in the FoxO3-NETT@SMs hydrogel group and the smoothness of the cartilage surface were even comparable to those of the sham group. Safranin O-Fast Green staining showed a similar trend, simultaneously reflecting the thickness of the stained cartilage. Compared to the PBS, SMs, and NETT@SMs treatment groups, the FoxO3-NETT@SMs hydrogel treatment group displayed a more uniform positive staining cartilage layer, with cartilage thickness closer to that of the Sham group (Fig. 7F). Moreover, the severity of joint damage was evaluated based on staining results using the OARSI score, as shown in Supporting Information Fig. S13. In DMM rats, the FoxO3-NETT@SMs treatment group had the lowest scores, indicating the most intact articular cartilage structure and the least extent of damage.

At 8- and 12-weeks post-surgery, immunohistochemical staining was conducted to assess the expression of cartilage specific matrix such as COL2, AGG, and COL10. According to the staining results (Fig. 7C and D), in the COL2 and AGG staining images, the FoxO3-NETT@SMs treatment group and sham group exhibited deep brown staining with large colored areas, while the PBS, SMs, and NETT@SMs treatment groups displayed light brown staining with smaller colored areas, indicating that the expression of cartilage-related matrix proteins is highest in the FoxO3-NETT@SMs group. However, in the COL10 immunohistochemical staining results, the FoxO3-NETT@SMs treatment group and sham group exhibited light brown staining, while the other samples showed dark brown staining (Fig. 7E). Semi-quantitative statistical results in Fig. 7G–I corroborated the immunohistochemical staining images results. The tissue staining results suggested that FoxO3-NETT@SMs hydrogel could mitigate OA progression through the secretion of cartilage-related proteoglycans, thus preventing cartilage degradation under OA conditions.

### 3.7 Expression of different mRNAs reveals molecular mechanism of FoxO3-NETT@SMs in OA therapy

We used transcriptomic analysis to determine the mechanisms involved in FoxO3-NETT@SMs enhancing ECM synthesis and cartilage regeneration at 8 weeks. To assess the stability of PBS, NETT@SMs, and FoxO3-NETT@SMs groups, Pearson correlation and principal component analysis were performed (Supporting Information Figs. S14 and S15). Results indicated acceptable correlation coefficients within an *R*^2^ > 0.95 range (*n* = 3), suggesting good biological reproducibility and usability of the transcriptomic data for further analysis. Differential gene expression analysis was conducted using volcano plots (Supporting Information Fig. S16). An analysis was conducted between the FoxO3-NETT@SMs and NETT@SMs groups (Fig. S16A), revealing the therapeutic impact of FoxO3 gene-editing on OA chondrocytes. This analysis identified 460 upregulated and 506 downregulated differentially expressed genes (DEGs). A separate comparison between the NETT@SMs and PBS groups (Fig. S16B) demonstrated the combined influence of the oxidative stress microenvironment regulation ability and enhanced lubrication of NETT@SMs microspheres, with 391 upregulated and 278 downregulated DEGs.

**Figure 8:**
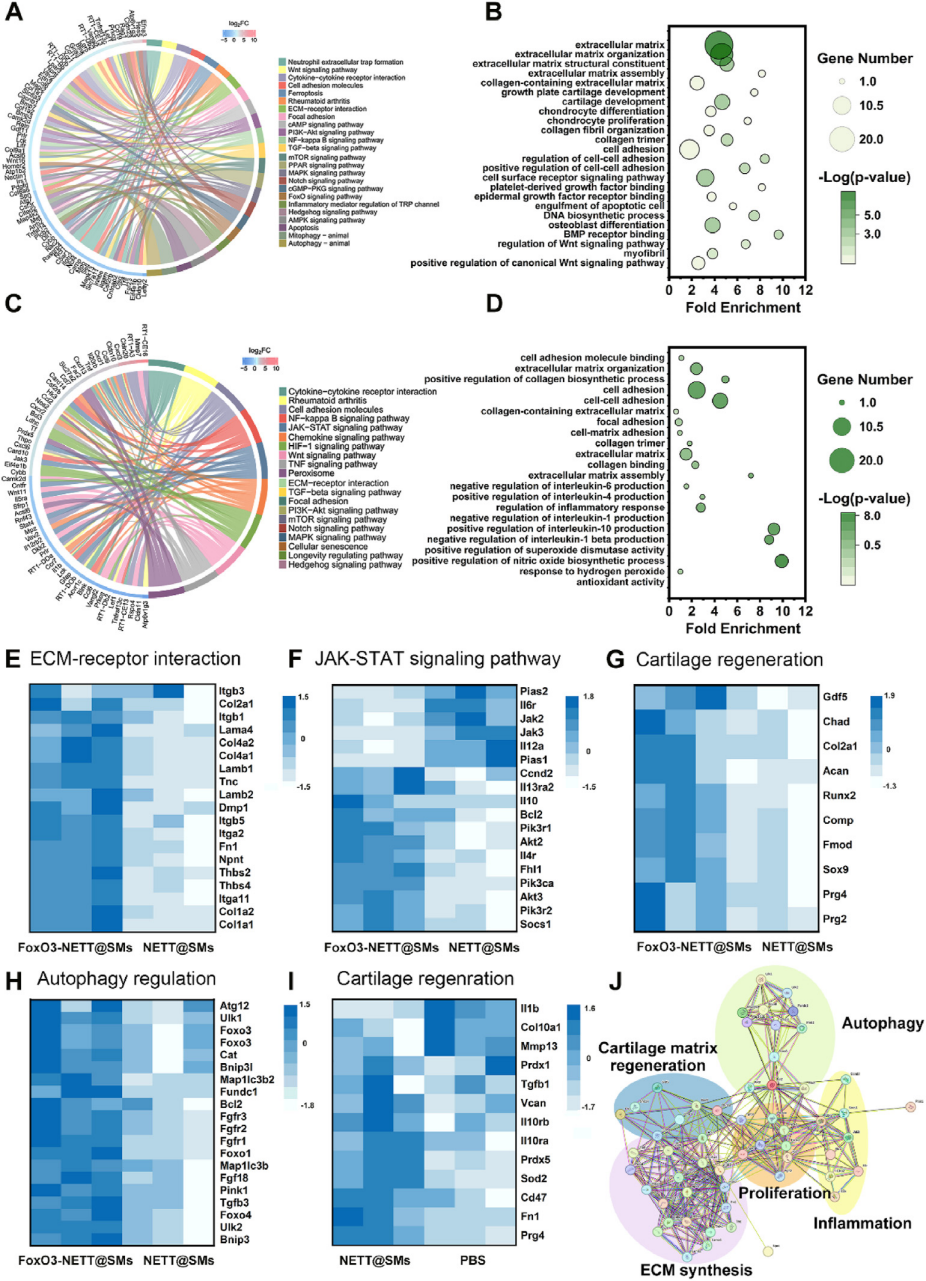
Differentially expressed mRNA comparison revealed osteoarthritis therapy mechanism *in vivo*. (A) Enriched KEGG terms of FoxO3-NETT@SMs groups *versus* NETT@SMs groups. (B) Enriched up-GO terms of FoxO3-NETT@SMs groups *versus* NETT@SMs groups. (C) Enriched KEGG terms of NETT@SMs groups *versus* PBS groups. (D) Enriched up-GO terms of NETT@SMs groups *versus* SMs groups. Heatmap of differentially expressed mRNA of FoxO3-NETT@SMs groups *versus* NETT@SMs groups involved in (E) ECM-receptor interaction, (F) JAK-STAT signaling pathway, (G) cartilage regeneration, and (H) autophagy regulation. (I) Heatmap of differentially cartilage regeneration associated expressed mRNA of NETT@SMs groups *versus* PBS groups. (J) String interaction network of differentially expressed proteins between FoxO3-NETT@SMs groups *versus* NETT@SMs groups. *n* = 3, independent experiments per group.

Kyoto Encyclopedia of Genes and Genomes (KEGG) pathway enrichment and GO enrichment analyses were conducted for differentially expressed genes. To investigate the effect of FoxO3 gene-editing in OA chondrocytes, KEGG pathway analysis of FoxO3-NETT@SMs *versus* NETT@SMs revealed top enrichment in ECM-receptor interaction, inflammation regulation (AMPK signaling pathway and NF-*κ*B signaling pathway), cell adhesion (cell adhesion molecules and focal adhesion), cell signaling and cellular communication (PI3K-AKT signaling and TGF-beta signaling pathway) and cell autophagy regulation (FoxO signaling, mitophagy-animal and autophagy-animal) (Fig. 8A). For GO database analysis of FoxO3-NETT@SMs and NETT@SMs (Fig. 8B), upregulated genes were involved in the production or synthesis of the extracellular matrix, such as positive regulation of extracellular matrix structure constituent, extracellular matrix organization, and collagen-containing extracellular matrix. Moreover, they were associated with cell adhesion regulation, such as positive regulation of cell adhesion and cell surface receptor signaling pathway. Additionally, they were related to cartilage regeneration, such as growth plate cartilage development, cartilage development, chondrocyte proliferation and collagen fibril organization. To investigate the effects of biomaterials, combining the SA microspheres and enhanced lubrication, KEGG pathway analysis showed top enrichment of NETT@SMs *versus* PBS groups in cell signaling and communication (cell adhesion molecules, ECM-receptor interaction and cytokine–cytokine receptor interaction), cell survival and proliferation (PI3K-AKT signaling pathway and Wnt signaling pathway), and inflammation regulation (TGF-beta signaling pathway and NF-*κ*B signaling pathway) (Fig. 8C). For GO database analysis (Fig. 8D), upregulated genes participated in cell migration and adhesion, ECM synthesis, collagen biosynthesis, inflammatory response, response to hydrogen peroxide, antioxidant activity, and positive regulation of superoxide dismutase activity when comparing NETT@SMs to PBS.

The heatmap (Fig. 8E–H) displayed the relative gene expression levels independently for the three samples, confirming that FoxO3 gene-editing (FoxO3-NETT@SMs *versus* NETT@SMs) played a positive role in promoting ECM-receptor interaction (*Col2a1*, *Lamb1*, *Lamb2*, *Lama4* and *Itgb11*), regulating inflammation involved in the JAK-STAT signaling pathway (*Pias2*, *Jak2*, *IL10* and *IL4r*), and maintaining cartilage function and regeneration (*Gdf5*, *Runx2*, *Acan*, *Col2a1*, *Sox9*, *Chad*, *Comp*, *Prg4* and *Prg2*), and modulating autophagy (*Foxo3*, *Pink1*, *Bnip3*, *Bnip3I*, *Cat* and *UIK1*). Moreover, the biomaterial, combining the SA microspheres and enhanced lubrication (NETT@SMs *versus* PBS), played a crucial role in inhibiting cartilage matrix degradation (*Col10a1*, and *Mmp13*), mediating inflammation (*IL10rb*, *IL10ra* and *IL-1β*), regulating oxidative stress (*Sod2*, *Prdx1* and *Prdx5*), as well as lubricating and maintaining cartilage function (*Prg4*, *Tgfb1*, *Vcan*, *Fn1* and *CD47*) (Fig. 8I).

A protein–protein interaction network of FoxO3-NETT@SMs groups *versus* NETT@SMs groups (Fig. 8J) identified specific genes filtered from GO and KEGG terms, categorized into five typical gene groups: ECM synthesis, cell proliferation, inflammation, autophagy and cartilage matrix regeneration. A protein–protein interaction network of NETT@SMs groups *versus* PBS groups (Supporting Information Fig. S17) identified specific genes filtered from GO and KEGG terms, categorized into four typical gene groups: cell adhesion, inflammatory regulation, regulation of oxidative stress and cartilage homeostasis and lubrication.

To this end, the CRISPR/Cas9 based FoxO3 gene-editing tools in FoxO3-NETT@SMs can regulate autophagy and restore normal mitochondrial function. Concurrently, the effects of biomaterials in FoxO3-NETT@SMs, including the SA microspheres and enhanced lubrication attributed to the lipid-based boundary *via* the integration of NETT and SMs, help to reduce joint wear, promote cartilage matrix regeneration and secretion, and inhibit inflammatory response, which is conducive to the maintenance of cartilage homeostasis. According to the transcriptomic results *in vivo*, FoxO3-NETT@SMs could modulate the up-regulation of the *Foxo3*, an autophagy activation gene, in OA chondrocytes, reactivating mitochondrial autophagy, restoring mitochondrial dysfunction and cartilage microenvironment homeostasis in OA. It is evident that mitophagy activation is essential for impeding OA progress. However, the specific mechanisms underlying the autophagy activated by FoxO3-NETT@SMs remain to be understood.

### 3.8 FoxO3-NETT@SMs activate autophagy by modulating PINK1/Parkin pathway to restore mitochondrial dynamics balance

**Figure 9:**
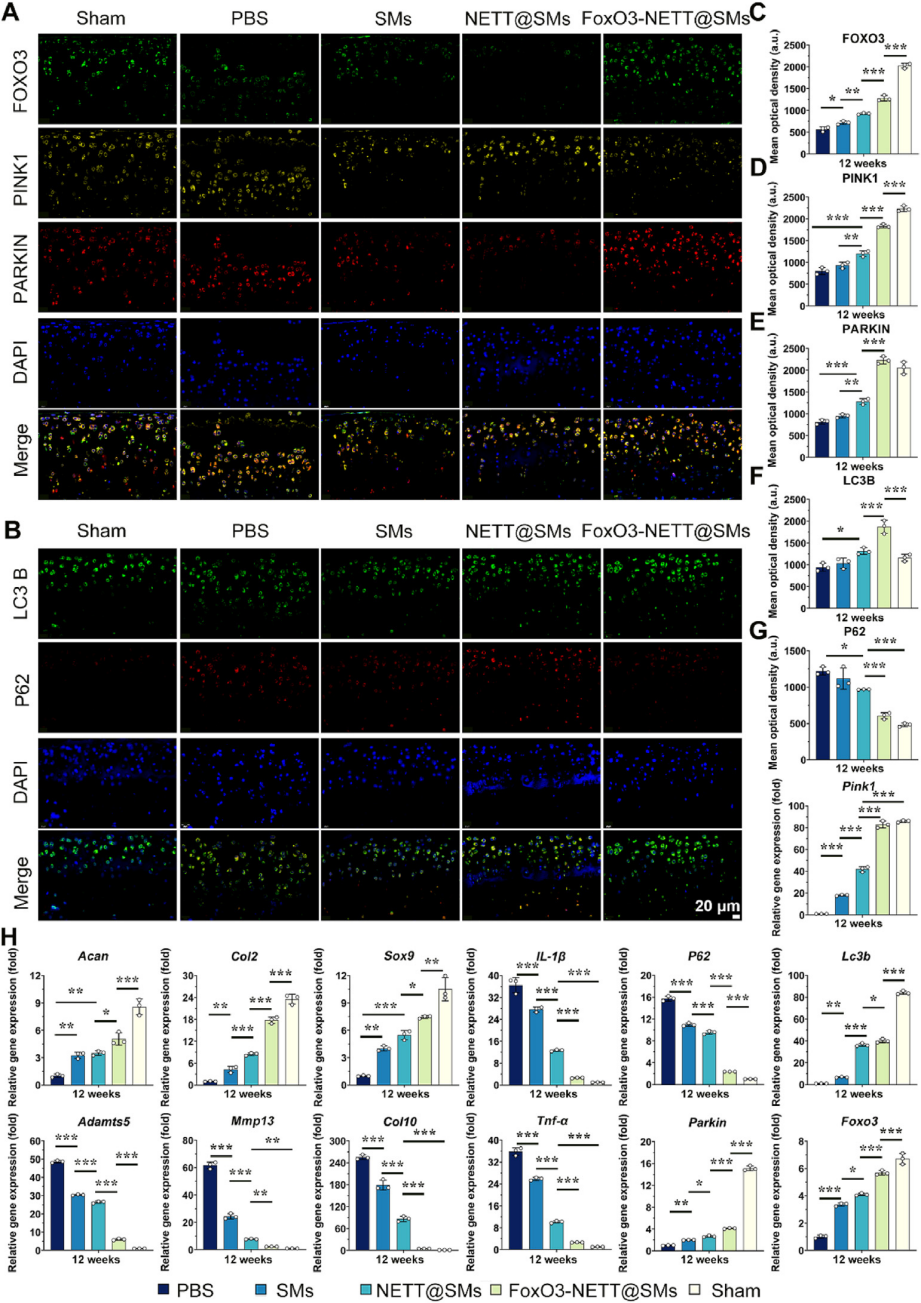
FoxO3-NETT@SMs activate autophagy by modulating PINK1/Parkin pathway to restore mitochondrial dynamics balance *in vivo*. Representative IF staining images of (A) FOXO3, PINK1 and PARKIN and (B) LC3B and P62 from each group after 12-weeks therapy. Scale bar = 20 μm. Semi-quantitative analysis of IF staining of (C) FOXO3, (D) PINK1, (E) PARKIN, (F) LC3B and (G) P62. (H) The genes expression related to cartilage ECM, fibrous and hypertrophic cartilage, matrix degradation, inflammatory regulation and autophagy regulation were detected by RT-PCR assay. *n* = 3, ∗*P* < 0.05, ∗∗*P* < 0.01, and ∗∗∗*P* < 0.001.

To elucidate the mechanism by which FoxO3-NETT@SMs activate autophagy, changes in the PINK1/Parkin signaling pathway were further investigated based on animal transcriptomic results *in vivo*. In the late stage of OA injury (12 weeks), the expression of FOXO3 in the transparent cartilage of the FoxO3-NETT@SMs treatment group significantly increased, while the PBS, SMs, and NETT@SMs groups all showed a significant decrease in FOXO3 expression. As anticipated, the *in-situ* delivery of FoxO3-NETT@SMs microspheres effectively upregulated FOXO3 levels in endogenous chondrocytes through FoxO3-NETT-mediated enhancement (Fig. 9A and C). Notably, immunofluorescence staining results from all groups at 12w revealed that upregulating FOXO3 could protect cartilage from OA damage, presumably by downregulating the expression of the apoptosis-related protein P62 (mtApoptosis) (Fig. 9B and G) and enhancing the expression of PINK1/Parkin-LC3B mitochondrial autophagy-related proteins (Fig. 9A, B and D–F), thus achieving a dynamic balance of mitochondrial dynamics and metabolic homeostasis *in vivo* in chondrocytes.

Furthermore, through RT-PCR testing of cartilage tissue samples from different groups, the expression levels of various genes were detected (Fig. 9H). Similarly, the *in-situ* delivery of “cargo” (FoxO3) by FoxO3-NETT@SMs microspheres, a switch regulating autophagy activation, downregulated the expression levels of the mitochondrial apoptosis-related gene *P62*, while enhancing the expression of mitochondrial autophagy-related genes *Pink1*, *Parkin*, and *Lc3b* through the upregulation of the *Foxo3* gene. By activating the mitochondrial autophagy pathway and inhibiting mitochondrial apoptosis, a dynamic balance of mitochondrial dynamics was achieved. While the limitation was that we were unable to collect sufficient specimens to extract cellular proteins and perform western blotting to detect the ratio of LC3 II to LC3 I, which would provide a more direct reflection of the generation of autophagic vesicles. Additionally, in the FoxO3-NETT@SMs treatment group, genes related to cartilage matrix regeneration and formation (*Acan*, *Col2*, and *Sox9*) were significantly upregulated, while genes associated with cartilage matrix metabolism and degradation (*Mmp13*, *Adamts5*, and *Col10*) were significantly suppressed, and inflammatory-related genes (*IL-1β* and *Tnf-α*) were significantly downregulated. This suggests that by mediating the upregulation of the *Foxo3* gene, restoring normal mitochondrial function, promoting cartilage matrix regeneration and secretion, and inhibiting the inflammatory response, FoxO3-NETT@SMs microspheres contribute to the maintenance of cartilage homeostasis. Collectively, FoxO3-NETT@SMs microspheres, through the activation of *Foxo3*, act as a switch for the autophagic PINK1/Parkin pathway. This involves mediating the accumulation of PINK1 on the outer mitochondrial membrane, activating Parkin, leading to the ubiquitination of damaged mitochondrial surface proteins^45^. LC3 then induces the fusion of autophagic vesicles with the damaged mitochondrial membrane, forming autophagosomes, which degrade the damaged mitochondria internally, completing the autophagic process^46^^,^^41^. This effectively clears damaged mitochondria, protecting damaged chondrocytes in OA and preventing further joint degeneration.

## 4 Discussion

Mitochondrial dysfunction in chondrocytes is a significant factor in OA. The enhancement of mitophagy and restoration of mitochondrial dynamics could potentially revitalize chondrocytes, thereby improving cartilage repair and regeneration. However, directly modulating mitochondria *in vivo* remains a significant challenge. This study pioneers the identification of a correlation between mitochondrial dysfunction and *FOXO3* downregulation across various OA stages in human cartilage, with further investigation in rodent OA joint tissues confirms a gradual, time-dependent decrease in FOXO3 protein expression as OA progresses to late stages, highlighting a potential therapeutic strategy for regulating mitochondrial dynamics *via FOXO3* gene modulation to alleviate OA.

Consequently, a novel nanoengineered ‘truck’ (NETT), loaded with a CRISPR/Cas9-based FoxO3-targeted gene-editing tool (Cas9-sgFoxO3), has been developed for *in vivo* targeting of *FOXO3*. The FoxO3-NETT, created by fusing SgFoxO3 plasmid-enriched liposomes with Cas9 protein-containing chondrocyte-targeting exosomes, is similar in size to exosomes and mildly positively charged, enhancing its targeting efficiency within the joint cavity. Compared to TExo, FoxO3-NETT exhibits improved stability over 30 days. *In vitro* studies confirm that FoxO3-NETT significantly boosts *Foxo3* gene expression in chondrocytes, demonstrating effective gene regulation and sustained cargo retention for autophagy regulation in OA chondrocytes.

The FoxO3-NETTs are further anchored within methacrylic acid-grafted sodium alginate and encapsulated into uniform hydrogel microspheres to form FoxO3-NETT@SMs. Studies show that only about 75% of FoxO3-NETTs are released after two weeks, suggesting an extended *in vivo* action time. The superior lubrication performance of FoxO3-NETT@SMs is confirmed, indicating benefits such as reduced friction and improved lubrication in the joint cavity. The results indicate that this nano-engineered microsphere-mediated gene therapy approach for OA demonstrates favorable *in vivo* stability and biocompatibility. The tribology findings confirm the superior lubrication performance of FoxO3-NETT@SMs, indicating that the intra-articular injection of FoxO3-NETT@SMs provides considerable benefits, including reduced friction, improved lubrication, and prevention of joint wear and tear within the joint cavity.

*In vitro* results show that FoxO3-NETT@SMs effectively reactivate mitochondrial autophagy, suppress mitochondrial apoptosis, and restore mitochondrial dysfunction and dynamic stability, further enhancing OA chondrocyte proliferation, migration, and ECM synthesis. *In vivo* data from a rat OA model validate the therapeutic potential of FoxO3-NETT@SMs to repair cartilage defects and alleviate OA progression. Transcriptomic analysis and further molecular validations elucidate the underlying mechanisms. FoxO3-NETT@SMs restore normal mitochondrial function by modulating the PINK1/Parkin autophagy signaling pathway, promoting cartilage matrix regeneration and secretion, and inhibiting inflammatory responses, thus favoring cartilage homeostasis.

## 5 Conclusions

In summary, this study revealed a connection between mitochondrial dysfunction and downregulation of *FOXO3* across various stages of human OA cartilage, suggesting a potential option for alleviating OA. To target *FOXO3 in vivo*, this study utilized microfluidic technology to combine nanoengineered vehicles (NETT) specifically targeting chondrocytes and loaded with “cargo” (FoxO3) – a switch regulating autophagy activation – with sodium alginate methacrylate (SM) to prepare UV-crosslinked injectable hydrogel microspheres (FoxO3-NETT@SMs). FoxO3-NETT@SMs effectively activated the expression of the FoxO3 gene in OA chondrocytes, achieving targeted enrichment and sustained retention of the “cargo” regulating autophagy activation switch in OA pathological cells. The utilization of CRISPR/Cas9 gene editing technology achieves precise modulation of *FOXO3* expression, associated with mitochondrial autophagy regulation. This allows for direct, precise *in vivo* targeting and delivery of biomolecules to cells, thereby ensuring biological safety. These findings suggest that FoxO3-NETT@SMs could serve as a promising therapeutic approach for OA patients. This study also highlights the advancement of CRISPR/Cas9-based gene-editing strategies for OA treatment, showcasing the potential of nanoengineered cargo with targeted *in vivo FOXO3* gene regulation to modulate mitochondrial dynamics in chondrocytes for effective OA intervention.

## Author contributions

Manyu Chen: Writing – original draft, Investigation, Data curation, Conceptualization. Yuan Liu: Writing – original draft, Investigation, Data curation, Conceptualization. Quanying Liu: Methodology, Formal analysis. Siyan Deng: Investigation, Data curation. Yuhan Liu: Methodology. Jiehao Chen: Investigation. Yaojia Zhou: Methodology. Xiaolin Cui: Writing – review & editing. Jie Liang: Validation. Xingdong Zhang: Supervision. Yujiang Fan: Writing – review & editing, Resources. Qiguang Wang: Writing – review & editing, Writing – original draft, Supervision, Resources, Conceptualization. Bin Shen: Writing – review & editing, Resources, Conceptualization.

## Conflicts of interest

The authors have no conflicts of interest to declare.

## Appendix A Supporting information

The following is the Supporting data to this article:
