# Supplementary material for: Nanoengineered cargo with targeted in vivo Foxo3 gene editing modulated mitophagy of chondrocytes to alleviate osteoarthritis
Source: Acta Pharm Sin B. 2024 Dec 14;15(1):571–91. doi: 10.1016/j.apsb.2024.12.008 (PMC11873664; doi:10.1016/j.apsb.2024.12.008)
Supplement: Multimedia component 1 [file mmc1.pdf]

Supporting Information for

ORIGINAL ARTICLE

Nanoengineered cargo with targeted *in vivo Foxo3* gene editing modulated mitophagy of chondrocytes to alleviate osteoarthritis

Manyu Chen[a,†], Yuan Liu[b,†], Quanying Liu[c], Siyan Deng[a], Yuhan Liu[d], Jiehao Chen[e], Yaojia Zhou[f], Xiaolin Cui[f,g], Jie Liang[a,h], Xingdong Zhang[a], Yujiang Fan[a]*, Qiguang Wang[a,]*, Bin Shen[b,]*

[a]*National Engineering Research Center for Biomaterials, College of Biomedical Engineering, Sichuan University, Chengdu 610064, China*

[b]*Orthopedics Research Institute, Department of Orthopedics, West China Hospital, Sichuan University, Chengdu 610041, China*

[c]*Institute of Rocket Force Medicine, State Key Laboratory of Trauma, Burns and Combined Injury, Third Military Medical University (Army Medical University), Chongqing 400038, China*

[d]*The Third Affiliated Hospital of Jinzhou Medical University, Jinzhou 121000, China*

[e] *Animal Laboratory Center of West China Hospital, West China Hospital, Sichuan University, Chengdu 610041, China*

[f]*Christchurch Regenerative Medicine and Tissue Engineering (CReaTE) Group, School of Medicine, the Chinese University of Hong Kong, Shenzhen 518172, China*

[g]*Department of Orthopedic Surgery & Musculoskeletal Medicine, Centre for Bioengineering & Nanomedicine, University of Otago, Christchurch 8140, New Zealand*

[h]*Sichuan Testing Center for Biomaterials and Medical Devices, Sichuan University, Chengdu 610064, China*

Received 11 July 2024; received in revised form 28 September 2024; accepted 10 October 2024

*Corresponding author.

E-mail addresses: fan_yujiang@scu.edu.cn (Yujiang Fan), wqgwang@126.com (Qiguang Wang), shenbin_1971@163.com (Bin Shen).

†These authors made equal contributions to this work.

1. Supporting figures

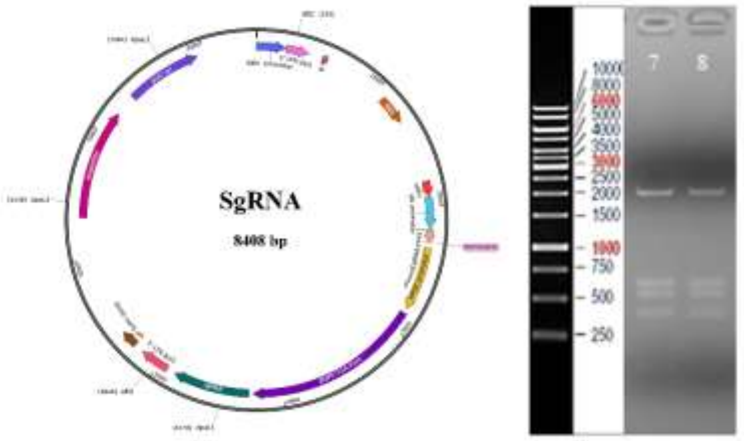

Figure S1 The plasmid DNA profiles of SgRNA (Left), results of nucleic acid sequence identified by sequencing (right).

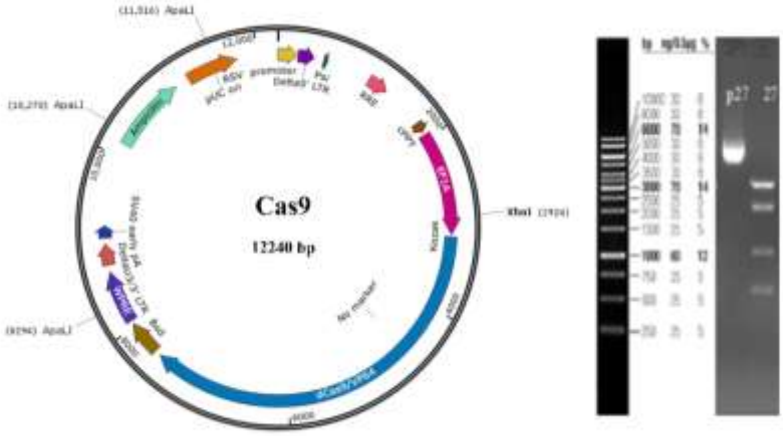

Figure S2 The plasmid DNA profiles of Cas9 (Left), results of nucleic acid sequence identified by sequencing (right).

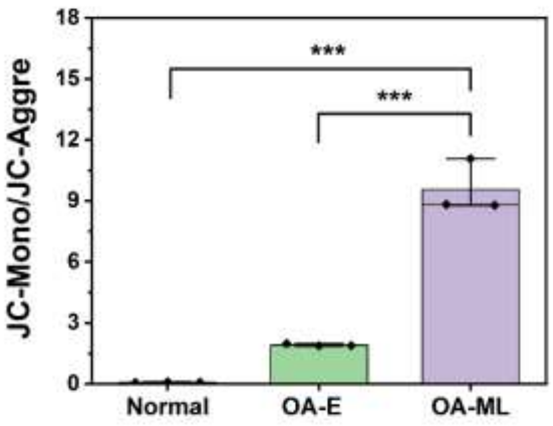

Figure S3 Semi-quantitative analysis of ratio of JC-Mono to JC-Aggre levels based on representative JC-1 staining images of cartilage isolated from patients with normal joints or from patients with early (OA- E) and middle (OA- ML) stage. n=3, ***P < 0.001.

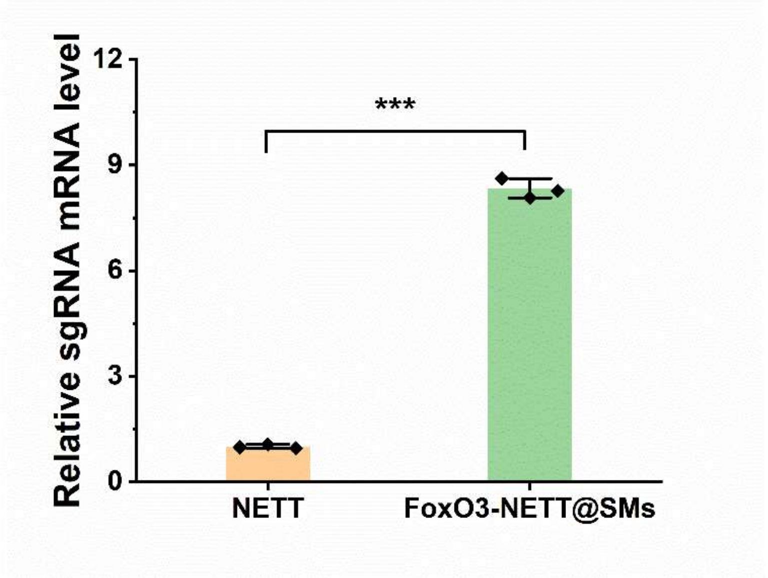

Figure S4 The sgRNA mRNA expressions in NETT and FoxO3-NETT was detected by qRT-PCR. n=3, ***P < 0.001.

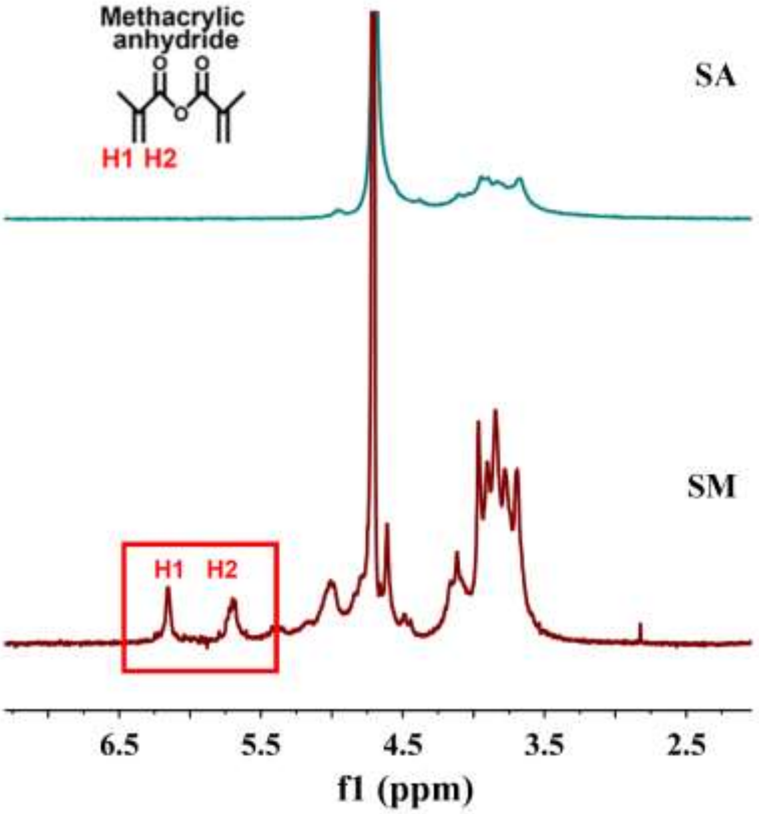

Figure S5 The ^{1}H NMR spectra of SA (top) and SM (below).

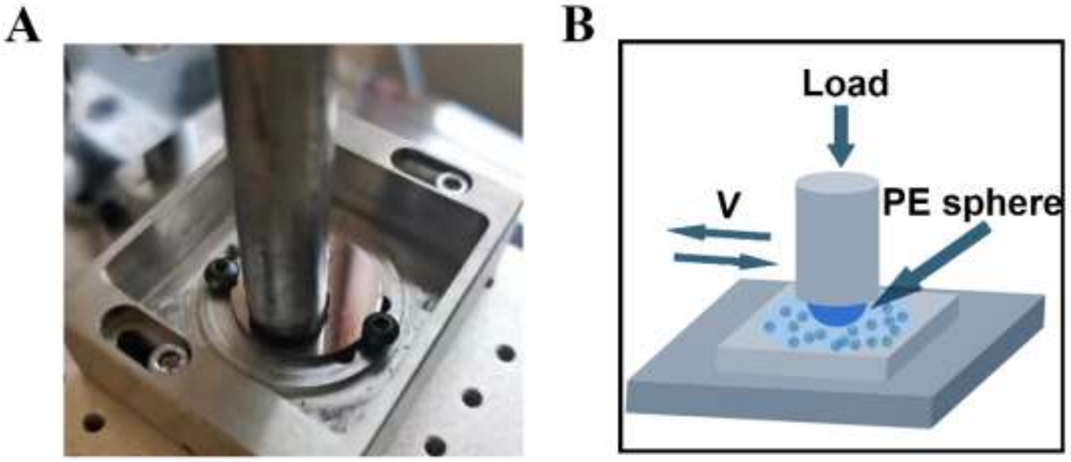

Figure S6 (A) Photograph and (B) schematic of the high-frequency reciprocating friction and wear testing.

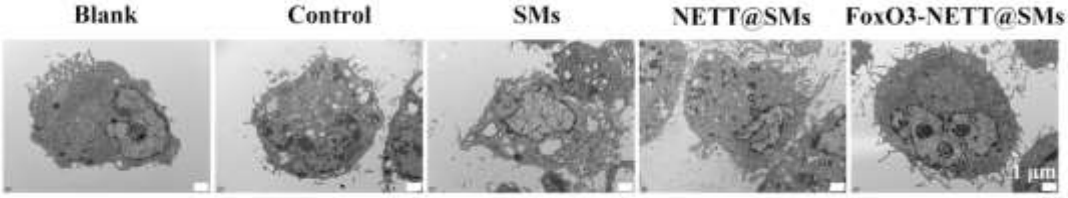

Figure S7 TEM images at lower magnification of OA chondrocytes (Blank), H_2O_2 treated OA chondrocytes (Control), and H_2O_2 treated OA chondrocytes cultured with SMs, NETT@SMs and FoxO3-NETT@SMs. Scale bar, 1 μm.

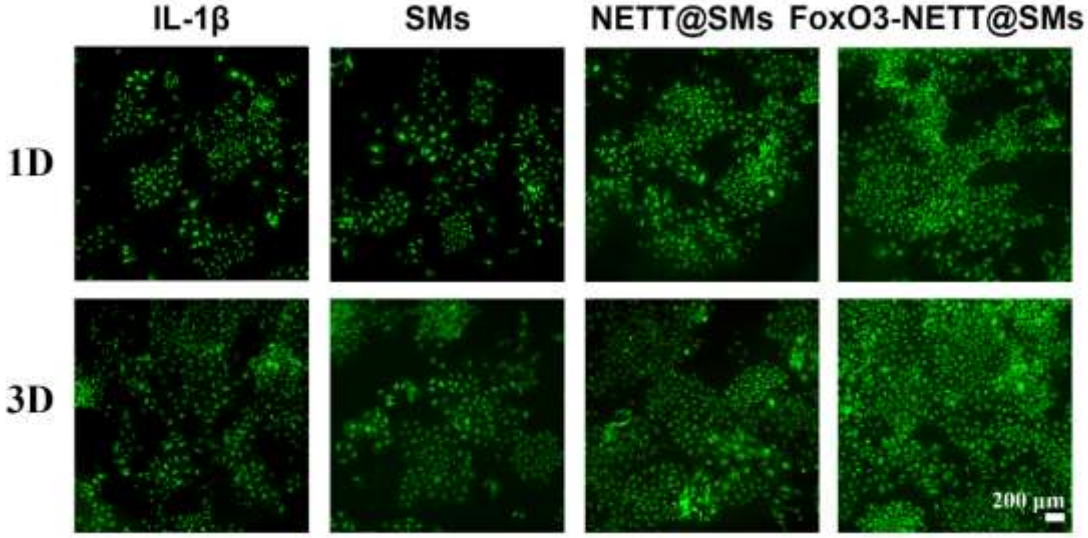

Figure S8 Cell death measured by Live/Dead assay after 1 and 3 days.

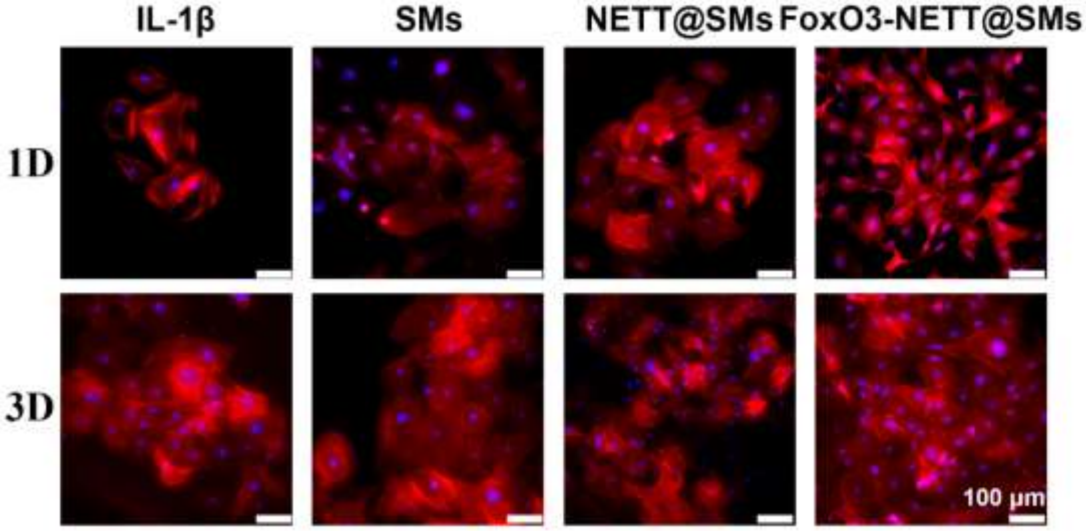

Figure S9 The cell cytoskeleton measured by phalloidin staining assay after 1 and 3 days.

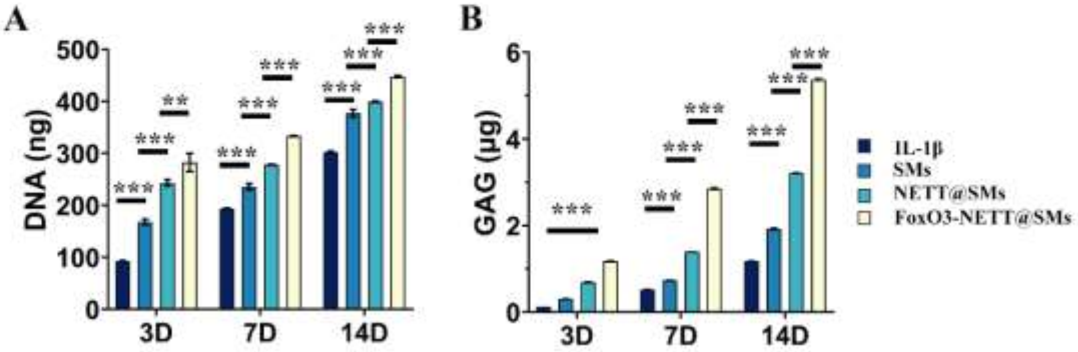

Figure S10 The results of DNA and GAGs of OA chondrocytes incubated with different samples after 3, 7 and 14 days. n=3, **$P < 0.01$, and ***$P < 0.001$.

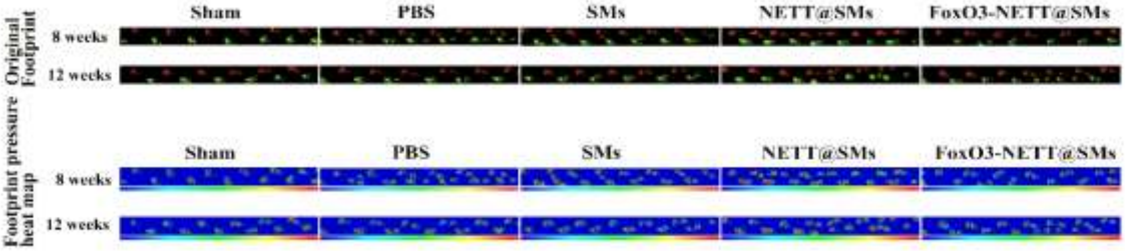

Figure S11 Representative gait screenshot, representative gait pattern images, and representative gait intensity images.

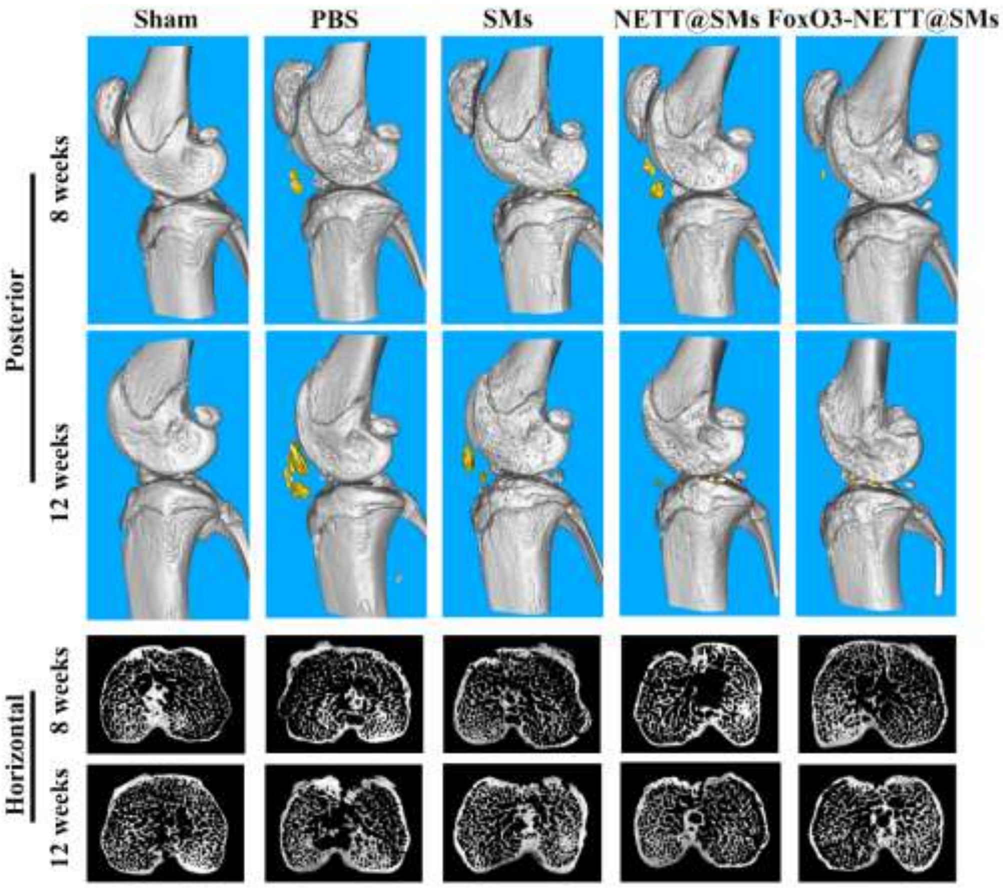

Figure S12 Representative Micro CT images after 8- and 12-weeks joint cavity injection.

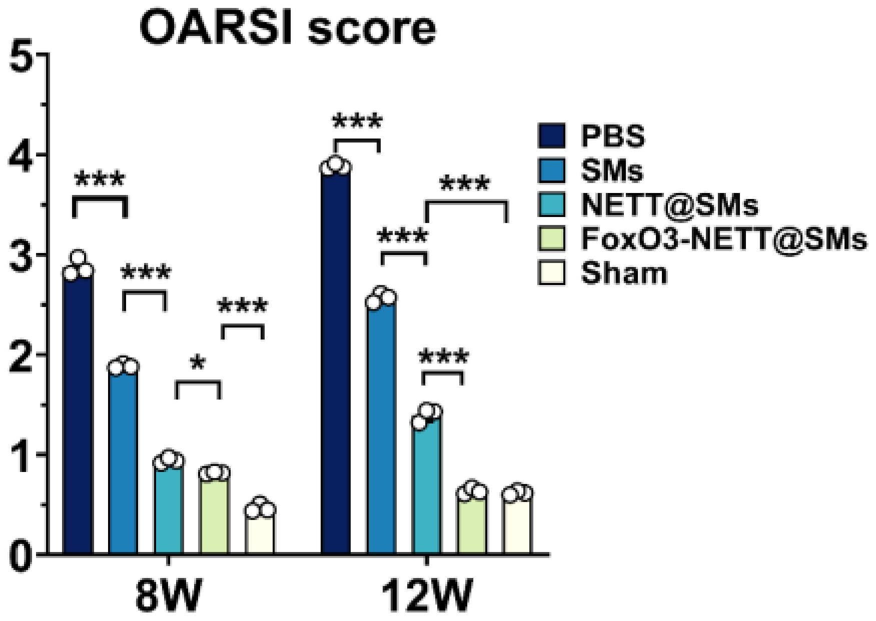

Figure S13 Cartilage OARSI score in Safranin O-fast green-stained sections. n=3, *P < 0.05 and ***P < 0.001.

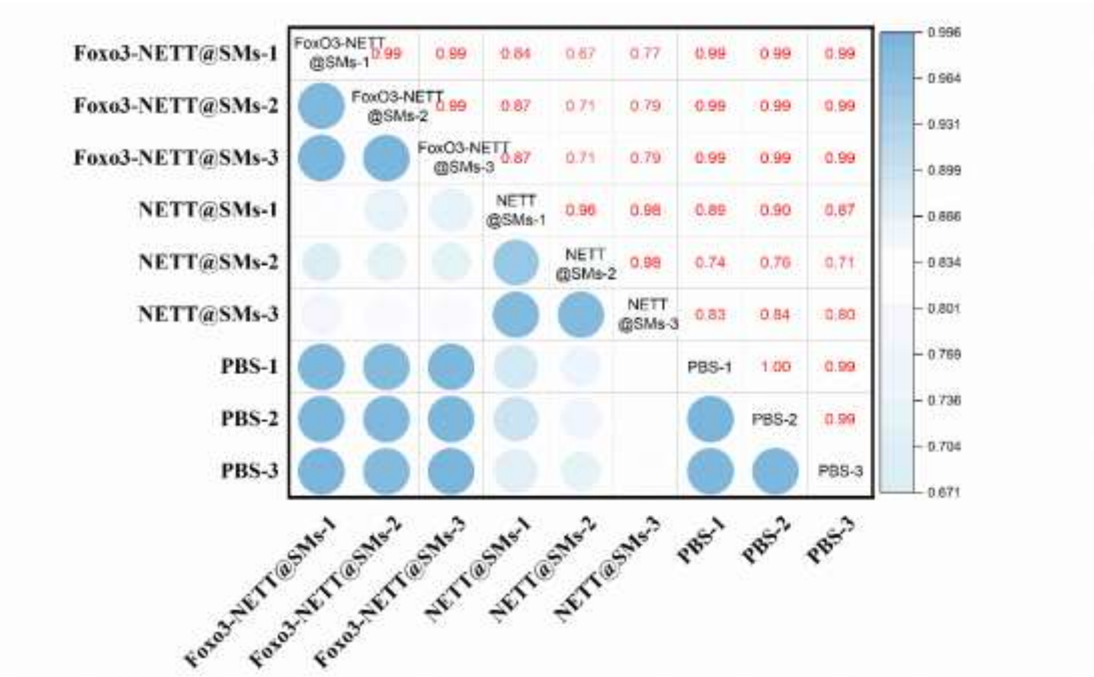

Figure S14 Correlation plot of different groups.

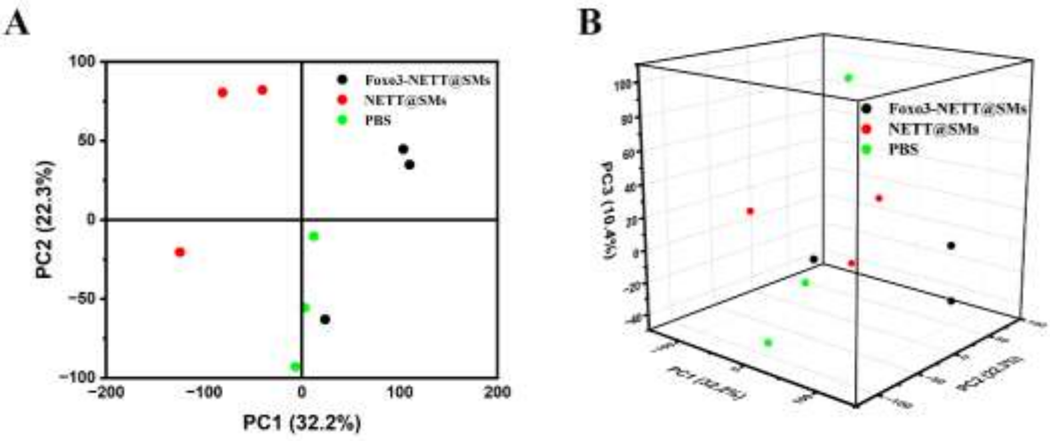

Figure S15 PCA analysis of different groups.

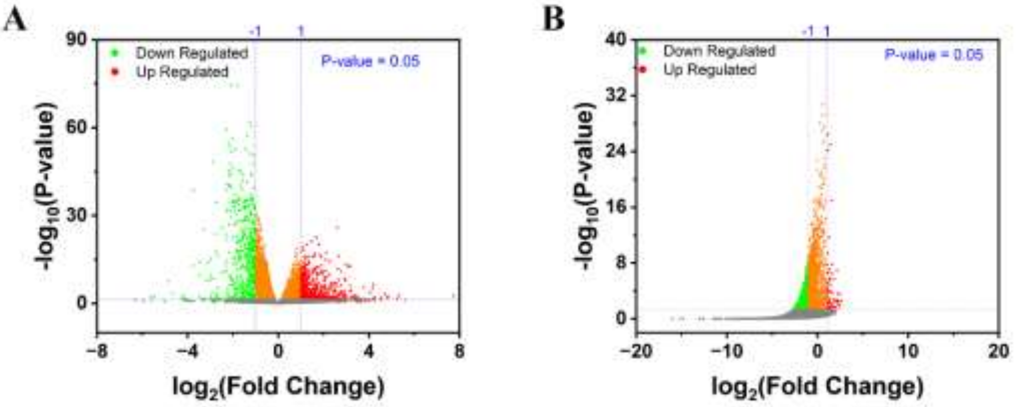

Figure S16 Volcano plot of transcriptomic analysis of differentially expressed mRNA between (A) FoxO3-NETT@SMs groups *versus* NETT@SMs groups, (B) NETT@SMs groups *versus* PBS groups.

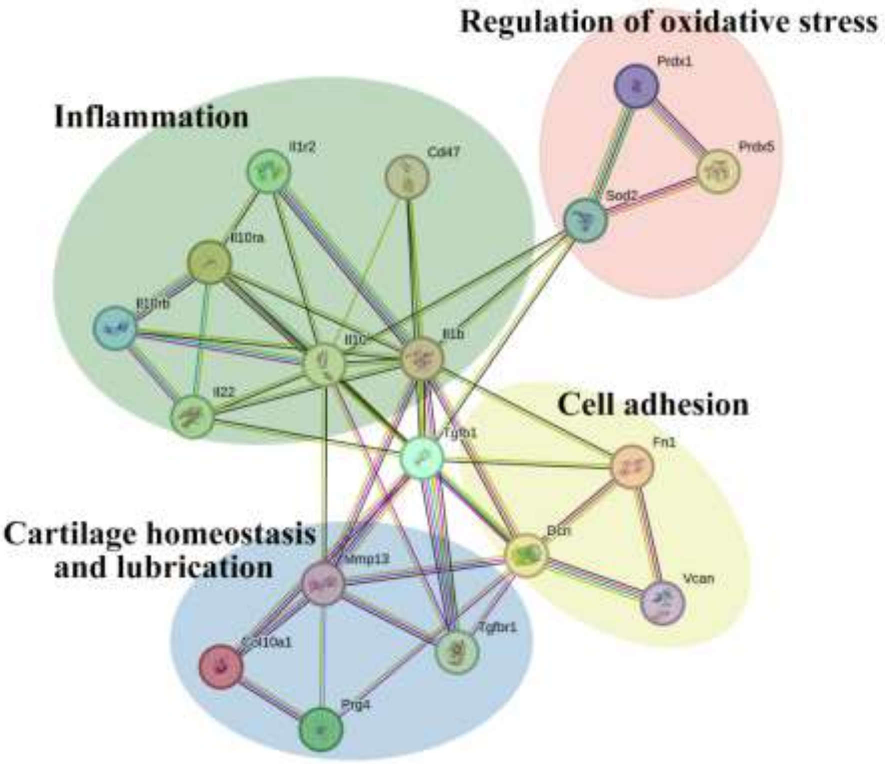

Figure S17 String interaction network of differentially expressed proteins between NETT@SMs groups *versus* PBS groups.

2. Supporting tables

Table S1 Primer sequences of Rattus norvegicus related genes.

Gene	Forward sequence	Reverse sequence
Gapdh	AGTGCCAGCCTCGTCTCATA	GGTAACCAGGCGTCCGATAC
Col2	ACGCTCAAGTCGCTGAACAACC	ATCCAGTAGTCTCCGCTCTTCC
Aggrecan	CTGATCCACTGTCCAAGCACCATG	ATCCACGCCAGGCTCCACTC
Sox9	TCAACGGCTCCAGCAAGAACAAG	CTCCGCCTCCTCCACGAAGG
Mmp13	AACCAAGATGTGGAGTGCCTGATG	CACATCAGACCAGACCTTGA AGGC
Adamts 5	TCCTCTTGGTGGCTGACTCTTCC	TGGTTCTCGATGCTTGCATG ACTG
Tnf-α	TTAGGAACGTGGATGAGACC	ATGCTGCTGTTCAGCTCTTC
IL-1β	CTCACAGCAGCATCTCGACAAGAG	TCCACGGGCAAGACATAGGTAGC
IL-10	CTGCTCTTACTGGCTGGAGTGAAG	TGGGTCTGGCTGACTGGGAAG
Col10	CAATGGTGAGGCAGGTCCAA	CCTGTTACCCCCTGGTTAGC
Lc3b	AAATAGAAGAGCAGTGTCAGGGG	GAGGTGAAATGGCTGGGGTT
Foxo3	AAGGGAGGAGGAGGAATGTGG	GTCAGCGTAGGACAGGTTCC
P62	ACTCTGCATTCTGGCTTCTGT	GACTTTACGGGGTGCCTCAA
Parkin	GAACTGTGGCTGTGAGTGGA	GGTGTTTCCCATGAGGTCGT
Pink1	GGAAAAGGCCCAGATGTCGT	GGAAAAGGCCCAGATGTCGT

Table S2 Primer sequences of human related genes.

Gene	Forward sequence	Reverse sequence
GAPDH	TGAAGGTCGGAGTCAACGGA	TTCCCGTTCTCAGCCATGTAGT
COL2	CCTGGCAAAGATGGTGAGACAG	CCTGGTTTTCCACCTTCACCTG
AGGRECAN	GACAAGCCAGGCGTGAGAA	ATCCACTCCTCCACACCAGA
SOX9	AGGAAGCTCGCGGACCAGTAC	GGTGGTCCTTCTTGTGCTGCAC
COL10	CGCTGAACGATACCAAATGCCC	TGGACCAGGAGTACCTTGCTCT
FOXO3	AAGGGAGGAGGAGGAATGTGGAA	GGTTGTGCCGGATGGAGTT
LC3B	AGCGTCTCCACACCAATCTC	CGGCGGGTTTTGTGAACCT
P62	TACCTTTGCCTCCACCTCCT	TCCCCTTGACTCTGGCTGTA
PARKIN	GGATTACCCAGGAGACCGCT	TCATGGTCACTGGGTAGGTG
